# Multi-matrix metabolomics in rare monogenic diabetes syndromes: Analysis of oral fluids and serum in carriers of pathogenic variants in the *ALMS1/BBS* genes

**DOI:** 10.1016/j.csbj.2025.10.040

**Published:** 2025-10-22

**Authors:** Ewa Zmysłowska-Polakowska, Patrycja Mojsak, Sebastian Skoczylas, Krzysztof Sołowiej, Sandra Chmielewska, Julia Grzybowska-Adamowicz, Aleksandra Palatynska-Ulatowska, Monika Lukomska-Szymanska, Adam Kretowski, Agnieszka Zmysłowska, Michał Ciborowski

**Affiliations:** a Department of Endodontics, Medical University of Lodz, Lodz, Poland; b Clinical Research Centre, Medical University of Bialystok, Bialystok, Poland; c Department of Clinical Genetics, Medical University of Lodz, Lodz, Poland; d Department of General Dentistry, Medical University of Lodz, Lodz, Poland; e Department of Medical Biochemistry, Medical University of Bialystok, Bialystok, Poland

**Keywords:** Alström syndrome, Bardet–Biedl syndrome, heterozygous carriers, oral metabolomics

## Abstract

Metabolomic profiling enables the identification of specific biochemical alterations in various diseases, including rare monogenic diabetes and obesity syndromes such as Alström syndrome (ALMS) and Bardet–Biedl syndrome (BBS). These disorders are characterized by early-onset obesity, insulin resistance, diabetes mellitus, retinodystrophy and other symptoms, but some features may also occur in heterozygous carriers of pathogenic or likely pathogenic variants in *ALMS1* and *BBS* genes. The aim of the study was to compare the metabolomic profiles of saliva, gingival crevicular fluid (GCF) and serum between ALMS/BBS patients and heterozygous carriers (n = 33) in relation to participants with simple obesity (n = 20) and healthy controls (n = 30) using gas chromatography coupled to mass spectrometry (GC-MS). The study showed significant differences in the levels of metabolites in saliva, GCF and serum, comparing the ALMS+BBS group with the other groups. In the analysis of statistically significant metabolites in all matrices, seven of them (valine, 3-HBA, alanine, threonine, urea, isoleucine, and phenylalanine) were consistently significant in all sample types. Levels of aromatic amino acids/branched-chain amino acids in saliva and serum were correlated with insulin resistance and the severity of obesity. In conclusion, the study identifies metabolic indicators that distinguish individuals with monogenic diabetes and obesity syndromes or genetic predisposition alone from other groups. The multi-matrix approach using available biological materials such as saliva and GCF provides a broader view of the metabolic alterations associated with variants in *ALMS1* and *BBS* genes. This approach may support both future mechanistic and translational studies of metabolic dysfunction in these populations.

## Graphical Abstract

**Figure:**
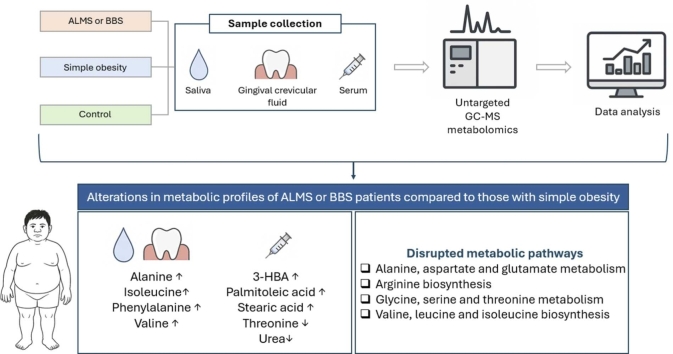

## 1 Introduction

Metabolomic studies conducted in recent years have expanded the knowledge of metabolites that affect organ function, immune processes, nutrient detection and intestinal physiology. Metabolomics focuses on the analysis of low-molecular-weight chemical compounds produced in cells, which act as signalling molecules in various physiological and pathophysiological processes [1], [2]. Numerous studies are underway on the use of metabolomic analysis in the development of diagnostic and prognostic biomarkers in many metabolic, oncological and cardiovascular diseases [3], [4], [5], [6]. Recent reports have identified metabolites in the blood that are associated with the pathogenesis of diabetes, the risk of developing diabetes and its complications, and the therapeutic response in both type 1 and type 2 diabetes [7], [8], [9]. Several studies also indicated a relationship between serum levels of selected metabolites and the presence of type 2 diabetes, insulin resistance and obesity in patients [10], [11], [12], [13], [14]. The importance of those findings should be considered in rare monogenic diabetes syndromes such as Alström syndrome (ALMS) and Bardet–Biedl syndrome (BBS), where type 2 diabetes coexists with early childhood obesity and insulin resistance.

Other common clinical features of ALMS and BBS include retinal dystrophy, hearing impairment, renal, cardiovascular, neurological or hepatic abnormalities, endocrinopathies, and bone defects [15], [16], [17]. These manifestations develop via a similar mechanism related to primary ciliary dysfunction. The two genetic syndromes are inherited in an autosomal recessive manner, and heterozygous carriers of variants in *ALMS1* or *BBS* genes may exhibit individual features associated with these disorders [16], [17], [18], [19], [20], [21]. Previous attempts to define progression markers of these syndromes using metabolomics have addressed the need for blood serum collection [22], [23]. However, reports indicate that more available biological materials, such as urine or saliva, can be used for metabolomic analysis in various monogenic diabetes syndromes [24], [25]. Nonetheless, comprehensive multi-matrix metabolomic investigations encompassing accessible fluids such as saliva and gingival crevicular fluid (GCF) remain scarce. The inclusion of these matrices could enhance the resolution of observable metabolic changes in these ciliopathies, offering a more integrated perspective of disease mechanisms. Gas chromatography coupled with mass spectrometry (GC-MS) has proven to be a powerful analytical tool in metabolomic studies due to its high sensitivity, reproducibility, and ability to separate and identify a wide range of metabolites, including amino acids, organic acids, carbohydrates, and fatty acids [26], [27]. GC-MS-based metabolomic profiling in the context of ALMS and BBS allows for the detection of subtle metabolic alterations associated with ciliary dysfunction and related systemic complications.

Therefore, the aim of this study was to evaluate the metabolomics profile of saliva and GCF obtained from patients with ALMS and BBS syndromes, as well as heterozygous carriers of variants in the *ALMS1* and *BBS* genes, using both a comparison group and a healthy control group, and to validate the results obtained in serum. In addition, selected metabolite–clinical parameter correlations were assessed within the study group.

## 2 Patients and methods

### 2.1 Study groups and protocol

The study protocol was approved by the University Bioethics Committee at the Medical University in Lodz, Poland (RNN/216/23/KE). Patients and/or their parents gave written informed consent for participation in the study.

The study group included 10 patients with genetically confirmed ALMS or BBS syndromes (3/7), as previously described [28], [29], and 23 heterozygous carriers of causative variants in *ALMS1* and *BBS* genes (10/13). The results were related to 20 patients in the comparison group with simple obesity (without accompanying symptoms), matched for age (*p* = 0.365) and BMI (Body Mass Index) (*p* = 0.209). The control group included 30 healthy individuals (without overweight/obesity, glucose metabolism disorders or chronic diseases) matched to the other groups in terms of age (*p* = 0.197). All patients in the study and comparison groups (patients with overweight or obesity) had a low-glycemic index and low-fat diet. No patient in the study, either in the comparison or control group, was diagnosed with periodontal disease. Detailed characteristics of the participants are presented in Table 1.

**Table 1:** Characteristics of the study groups. ALMS – Alströme syndrome; BBS – Bardet–Biedl syndrome; BMI – body mass index; SSD – standard deviation; F – female; M – male.

**Parameters**	**Study group (patients with ALMS+BBS and heterozygotes)**	**Comparative group (patients with simple obesity)**	**Control group**
	**Mean±SD**	**Mean±SD**	**Mean±SD**
Age at examination (year)	34.2 ± 14.0	42.3 ± 14.4	30.1 ± 14.0
Body weight (kg)	83.1 ± 24.3	88.1 ± 12.8	62.7 ± 10.2
BMI (kg/m^2^)	34.3 ± 6.4	30.5 ± 3.9	21.3 ± 2.9
N (F; M)	33 (21 F; 12 M)	20 (13 F; 7 M)	30 (22 F; 8 M)

The metabolomic profiling results were then compared with selected laboratory parameters, such as glucose metabolism parameters (glucose and insulin levels, HOMA-IR – Homeostatic Model Assessment for Insulin Resistance, glycated hemoglobin (HbA1c), lipids profile (total cholesterol, LDL, HDL levels and triglycerides –TG), renal function markers (serum creatinine and urine). The results were also compared with clinical data such as patient age, weight, height and BMI. The mean values ± standard deviation in the study group (ALMS+BBS) were: HbA1c 5.4 ± 0.7 %, total cholesterol 5.32 ± 1.1 mmol/l, HDL-cholesterol 1.38 ± 0.3 mmol/l, triglycerides 1.32 ± 0.7 mmol/l, and LDL-cholesterol 3.5 ± 0.8 mmol/l.

Saliva and GCF samples were collected from patients in the study, as well as from the comparison and control groups, in a non-invasive manner during a routine intraoral dental examination performed by two experienced dentists.

Saliva was collected between 8:00 a.m. and 10:00 a.m. All study participants were asked to refrain from eating, drinking, smoking and using oral hygiene procedures for eight hours prior to saliva collection. The samples were collected using the Salivette® diagnostic collection tube (Sarstedt, Germany) with a synthetic swab, which was held in the mouth by the patients for 2 min. The collected samples were transported on ice immediately after collection and clarified by centrifugation at 4°C (1000 x g for 5 min). The supernatant was stored at −80°C until metabolomic analysis.

Prior to GCF extraction, patients performed proper tooth brushing according to received instructions received. GCF was then collected using sterile PERIOPAPER™ absorbent strips (Oraflow Inc, NY, USA), as described previously [24]. In addition, for validation purposes, serum was obtained using a standard centrifugation procedure at 4°C (2000 x g for 10 min) and stored at −80°C for further analysis.

### 2.2 Metabolomics – reagents

The following reagents and standards were utilized in this study: Milli-Q® water (Millipore, Billerica, MA, USA), heptane (Sigma–Aldrich, Steinheim, Germany), pyridine (Sigma–Aldrich, Steinheim, Germany), and O-methoxyamine HCl (Sigma–Aldrich, Steinheim, Germany). Additionally, MSTFA (N-Methyl-N-trimethylsilyl-trifluoroacetamide) with 1 % TMCS (Trimethylchlorosilane) (Pierce Chemical Co., Rockford, IL, USA) was used. For GC–MS analysis, 4-nitrobenzoic acid (4–NBA) and stearic acid methyl ester (C18:0 methyl ester) (Sigma–Aldrich, Steinheim, Germany) were employed as internal standards (ISs). Individual stock solutions of 4–NBA (IS1) were prepared at a concentration of 25 ppm and stored at −4 °C, while methyl stearate (IS2) solutions were prepared at a concentration of 20 ppm and stored at −20 °C. Additionally, two standard mixtures for GC–MS analysis were purchased: one containing grain-derived fatty acid methyl esters (FAME) (C8:0—C22:1, n9) and another consisting of a mixture of n-alkanes (C8:C40) (Supelco, Bellefonte, PA, USA).

### 2.3 Metabolomics – sample preparation

Extraction of GCF metabolites was performed as described previously [24], while for the saliva and plasma samples, an aliquot of 40 µL of plasma/saliva and 120 µL of cold acetonitrile containing the IS1 (25 ppm) were mixed for metabolite extraction [30].

In the next step, 30 μL of O-methoxyamine hydrochloride (20 mg/mL) in pyridine was added to all the samples. The vials were closed and vortexed vigorously for 2 min, ultrasonicated for 5 min, and vortexed again for 2 min. The vials were covered with aluminum foil and incubated under darkness at room temperature for 16 h. After this time, 30 μL of MSTFA with 1 % TMCS was added to each sample. The vials were closed and vortexed for 2 min. The GC vials were placed into an oven for 1 h at 70°C for silylation, then cooled at room temperature in the dark for approximately 1 h. Subsequently, 60 μL of heptane containing 5 ppm of methyl stearate (MS) was added to each sample and vortexed for 2 min. Quality control (QC) samples were independently prepared by pooling equal volumes of each sample and following the same extraction procedure as the experimental samples. An analyte-free extraction blank and a reagent blank were also processed.

### 2.4 Untargeted GC–MS data analysis

Metabolic fingerprinting was conducted using a GC system (7890B series) equipped with a 7693 A auto-sampler and a 7000D Mass Selective Detector (Agilent Technologies, Palo Alto, CA, USA). A 1 μL aliquot of the derivatized sample, including internal standards (ISs), was injected into a DB–5MS capillary GC column (30 m × 0.25 mm × 0.25 μm). Helium was used as the carrier gas at a constant flow rate of 1.0 mL/min. The injector temperature was maintained at 250 °C, with a split ratio of 1:10. The temperature program commenced at 60 °C with a 1-minute hold, followed by a ramp-up to 320 °C at a rate of 10 °C/min. The GC–MS transfer line, filament source, and quadrupole were set at 280 °C, 230 °C, and 150 °C, respectively. The electron ionization source operated at 70 eV, and the mass spectrometer functioned in full scan mode, covering a mass range of *m/z* 50–600 with a scan rate of 1.38 scans per second.

### 2.5 Raw GC–MS data processing

Deconvolution and compound identification were performed using Mass Hunter Quantitative Unknowns Analysis software (B.07.00, Agilent, Santa Clara, CA, USA). Data alignment was performed with Mass Profiler Professional software (version 13.0, Agilent, Santa Clara, CA, USA), while peak integration was conducted using Mass Hunter Quantitative Analysis software (version B.07.00, Agilent, Santa Clara, CA, USA). Compound identification relied primarily on accurate mass and product ion spectrum matching against an in-house library of authenticated standards, as well as reference libraries such as Fiehn’s and NIST 14.

To facilitate differential analysis of the metabolomics data, variables were filtered following the method described by Godzien et al. [31]. Missing values were imputed using the k-means nearest neighbor approach [32], with scripts developed in MATLAB 7.10 R2010a (MathWorks Inc., Natick, MA, USA). The list of annotated metabolites is provided in Table S1.

Prior to statistical analysis, the areas of clinical sample peaks were normalized to minimize instrument-related response variability. For statistical comparisons, the GCF matrix was normalized to the total signal, while normalization was performed for the saliva and serum samples using the intensity of IS2.

### 2.6 Statistical analysis

To evaluate data quality, filtered datasets were subjected to Principal Component Analysis (PCA), and the clustering of quality control (QC) samples was inspected (Figure S1). Multivariate analyses were performed using SIMCA-P + version 13.0.3.0 (Umetrics, Umeå, Sweden). To visualize group separations and assess model performance, Partial Least Squares Discriminant Analysis (PLS-DA) was conducted (Figure S2), and model robustness was confirmed using permutation tests.

For comparisons between two groups, the non-parametric Mann–Whitney *U* test was applied, with p-values adjusted for multiple testing using the False Discovery Rate (FDR) correction. The non-parametric Kruskal–Wallis ANOVA test was used to determine whether metabolites differed significantly among the three groups: the study group (patients with at least one causative variant in the *ALMS1* or *BBS* gene; Groups I and II), the comparison group (patients with simple obesity; Group III), and healthy controls (Group IV) (p < 0.05). When significance was observed, a post hoc pairwise comparison was conducted using the non-parametric Conover–Iman test (p < 0.05). Finally, Spearman’s rank correlation analysis (in R) was used to explore associations between metabolomic data and clinical characteristics (version 4.0.0).

To assess whether merging homozygous patients with heterozygous carriers was justified, we compared their metabolite profiles using non-parametric rank-biserial correlations. Effect sizes were calculated for metabolites distinguishing cases from controls and interpreted according to Cohen’s conventions (Figures S3–S5).

Metabolomic data were analyzed using global ANOVA to assess differences across the studied groups. The Omega squared (ω²) statistic was calculated as a measure of global effect size, with corresponding 95 % confidence intervals (ω²_low and ω²_high) computed to estimate the precision of the effect. For pairwise comparisons between groups (IV vs III, IV vs ALMS+BBS, and III vs ALMS+BBS), Cohen’s d was calculated as a measure of effect size, along with 95 % confidence intervals (CI) (CI (low) and CI (high)) to assess the reliability of the observed differences. Non-parametric effect sizes were used where assumptions of normality were not met. This analysis was performed using the R software environment (version 4.0.0).

## 3 Results

We performed GC-MS analyses of saliva, GCF, and serum, detecting a total of 301, 158, and 382 raw peaks in the respective samples. After data preprocessing (including deconvolution, alignment, normalization, and filtering), 108, 36 and 101 metabolites were identified in saliva, GCF, and serum, respectively. These included several derivatives of individual metabolites, particularly amino acids and carbohydrates. All identified metabolites showed a relative standard deviation (RSD) of less than 30 %. In addition, the clustering of QC samples in the PCA plot (Figure S1) further confirmed the reproducibility of the analyses.

A series of statistical analyses was then performed for the following:

I) ALMS + BBS,
II) ALMS_H vs. BBS_H (heterozygous carriers of 1 pathogenic or likely pathogenic variant in *ALMS* and *BBS* genes),
III) Obesity (OB),
IV) Control group (Ctrl).

In the initial comparison, no statistically significant differences (p > 0.05) were observed between the following subgroup pairs: I ALMS versus I BBS, II ALMS versus II BBS, and Group I (I ALMS + I BBS) versus Group II (II ALMS + II BBS) in GCF and saliva samples, except for serum samples. Although some metabolites showed statistical significance, evaluation of percentage change and RSD indicated that analytical variability exceeded biological variability. Despite the distinct genetic backgrounds of these syndromes, both ALMS and BBS share a common pathological mechanism involving damage to primary ciliary structures, resulting in similar clinical manifestations. Furthermore, the similarity in obesity levels, as measured by BMI, along with the absence of statistically significant differences in participant age and metabolomic analysis results between subgroups I and II, justified their combination into a single study group (ALMS/BBS). This approach was intended to enhance the statistical power of the study, given the rarity of these genetic syndromes.

To ensure that merging homozygous patients with heterozygous carriers did not introduce bias, we calculated non-parametric effect sizes (rank-biserial correlations) for the main metabolites distinguishing cases from controls. Most effect sizes were below 0.3, indicating small effects, with a few metabolites showing values below 0.5, consistent with moderate effects according to Cohen’s conventions (see Figures S3–S5).

Figure S2 shows the clustering of samples among the three groups (study, comparison, and control), indicating a partial overlap between the groups of patients with OB and Ctrl, particularly in panels B (saliva) and C (serum), while in panel A the separation of groups is more distinct. This overlap highlights the common metabolic features in the comparison and control groups, emphasizing that their division is not absolute. In contrast, the study group (red) remains clearly distinct, confirming its specific metabolic signature. Among the analysed matrices, GCF (panel A) demonstrated the strongest group separation and the most robust model performance in both PLS-DA and permutation tests, indicating its superior discriminatory power and supporting its potential clinical and translational relevance.

Based on the comparison of the ALMS+BBS group with the obesity and control groups, changes were observed in the levels of 33, 19 and 34 metabolites in saliva, GCF, and serum samples, respectively ( Table 2, Table S4, Table S5). Most of these changes in metabolite levels in ALMS and BBS, relative to the patients in the OB and Ctrl groups, were related to amino acids, fatty acids, and carbohydrates across all matrices analyzed.

**Table 2:** Summary of statistical analysis (ANOVA/Kruskal–Wallis test) for saliva metabolite levels across study groups. Statistically significant p-values are in bold. Effect size metrics and 95 % confidence intervals for group comparisons. ω² – global ANOVA ω²; ω²_ci_low / ω²_ci_high – 95 % CI bounds for ω²; effsize – Cohen’s d for pairwise comparisons (IV vs III, IV vs ALMS+BBS, III vs ALMS+BBS); conf.low / conf.high – lower and upper 95 % CI bounds for each Cohen’s d.

**Subclass**	**Metabolites**	**HMDB**	**CV**	**ALMS+BBS vs. III vs. IV**	**Omega2**	**Omega2_ci_low**	**Omega2_ci_high**		**post-hoc**													
								**IV vs. III**	**% of changes**	**Effect size_IV vs. III**	**CI (low)_IV vs. III**	**CI (H)_IV vs. III**	**IV vs. ALMS+BBS**	**% of changes**	**effsize_IV vs. ALMS+BBS**	conf.low_IV vs. ALMS+BBS	conf.high_IV vs. ALMS+BBS	**III vs. ALMS+BBS**	**% of changes**	effsize_III vs. ALMS+BBS	conf.low_III vs. ALMS+BBS	conf.high_III vs. ALMS+BBS
Alcohols and polyols	Quinic acid	HMDB0003072	11.5	5.20E−02	0.00E+00	0.00E+00	9.38E−02	1.92E−01	−78.9	2.37E−01	−0.6	0.48	**1.80E−02**	**−96.3**	**0.3**	0.12	0.61	**6.14E−03**	**−86.1**	0.54387	0.17	0.94
	Scyllo-Inositol	HMDB0006088	16.9	**1.66E−02**	**1.53E−02**	**0.00E+00**	**1.53E−01**	1.98E−01	1.9	−1.83E−02	−0.5	0.81	**6.94E−04**	**−43.8**	**0.6**	0.08	1.23	**1.87E−02**	**−46.5**	0.40981	−0.14	0.89
Amines	Putrescine	HMDB0001414	7.3	9.77E−02	6.25E−03	0.00E+00	1.94E−01	4.01E−01	17.1	−9.12E−02	−0.56	0.56	**2.88E−02**	**103.0**	**−0.5**	−0.97	−0.02	**1.52E−02**	**149.7**	−0.3127	−1.04	0.24
	Cadaverine	HMDB0002322	6.9	5.26E−02	7.35E−02	1.29E−02	2.68E−01	3.52E−01	27.8	−1.48E−01	−0.6	0.63	**8.24E−03**	**199.0**	**−0.7**	−1.08	−0.31	**1.28E−02**	**165.3**	−0.53543	−1.06	−0.0074
	1,3-diaminopropane	HMDB0000002	18.4	**1.91E−03**	**1.10E−01**	**6.29E−02**	**3.88E−01**	1.58E−01	20.9	−1.91E−01	−0.79	0.33	**2.39E−05**	**672.0**	**−0.6**	−1.2	−0.43	**3.75E−03**	**448.2**	−0.55037	−1.16	−0.41
Amino acids, peptides and analogues	Alanine	HMDB0000161	9.4	**6.80E−03**	**1.37E−01**	**3.60E−02**	**3.08E−01**	4.37E−01	11.6	−1.32E−01	−0.67	0.39	**4.41E−04**	**118.1**	**−0.9**	−1.34	−0.45	**1.76E−03**	**106.3**	−0.78249	−1.28	−0.35
	Valine	HMDB0000883	7.5	**3.76E−03**	**6.24E−02**	**7.92E−03**	**2.95E−01**	2.96E−01	67.8	−2.81E−01	−0.61	0.5	**4.16E−04**	**207.4**	**−0.9**	−1.32	−0.58	**4.92E−04**	**175.1**	−0.36556	−1.2	0.15
	Proline	HMDB0000162	22.1	2.25E−01	0.00E+00	0.00E+00	1.51E−01	2.53E−01	−9.6	5.36E−02	−0.49	0.59	7.10E−02	61.3	−0.3	−0.82	0.22	**4.78E−02**	**165.0**	−0.31989	−0.92	0.16
	Isoleucine	HMDB0000172	7.0	**1.66E−02**	**4.31E−02**	**3.58E−03**	**2.69E−01**	3.31E−01	77.1	−3.15E−01	−0.61	0.48	**1.56E−03**	**187.9**	**−0.9**	−1.26	−0.49	**2.36E−03**	**135.0**	−0.26448	−1.16	0.24
	Serine	HMDB0000187	6.3	1.26E−01	9.26E−02	1.55E−02	2.41E−01	4.81E−01	−3.4	4.10E−02	−0.53	0.6	**2.82E−02**	**112.9**	**−0.7**	−1.06	−0.34	**3.19E−02**	**116.7**	−0.70115	−1.08	−0.33
	Threonine	HMDB0000167	6.6	**1.66E−02**	**1.17E−01**	**3.05E−02**	**2.88E−01**	4.56E−01	4.6	−5.93E−02	−0.68	0.47	**2.68E−03**	**121.0**	**−0.7**	−1.13	−0.38	**3.04E−03**	**115.0**	−0.72278	−1.1	−0.36
	Beta-alanine	HMDB0000056	6.6	**3.68E−02**	**4.82E−02**	**0.00E+00**	**2.29E−01**	2.91E−01	68.1	−3.32E−01	−0.69	0.29	**2.98E−03**	**158.3**	**−0.7**	−1.11	−0.39	**2.20E−02**	**147.1**	−0.32779	−0.97	0.18
	3-aminoisobutyric acid	HMDB0003911	4.9	**1.66E−02**	**2.59E−02**	**0.00E+00**	**1.53E−01**	9.05E−02	37.2	−5.72E−01	−1.09	−0.06	**5.72E−04**	**178.8**	**−0.5**	−0.89	−0.1	5.45E−02	104.8	−0.04571	−0.55	0.54
	Trans−4-hydroxy-L-proline	HMDB0000725	5.4	9.63E−02	6.58E−02	1.68E−03	2.04E−01	3.95E−01	3.8	−3.75E−02	−0.68	0.49	**2.81E−02**	**152.5**	**−0.6**	−0.91	−0.25	**1.42E−02**	**165.8**	−0.55788	−0.88	−0.24
	Glutamic acid	HMDB0000148	4.2	**2.77E−02**	**1.23E−01**	**3.28E−02**	**3.02E−01**	4.18E−01	20.6	−4.47E−01	−1.09	0.11	**3.01E−03**	**216.7**	**−0.8**	−1.15	−0.48	**8.72E−03**	**128.1**	−0.62159	−1	−0.19
	Gamma-aminobutyric acid (GABA)	HMDB00112	9.5	2.25E−01	0.00E+00	0.00E+00	1.92E−01	4.42E−01	99.0	−3.16E−01	−0.66	0.55	7.20E−02	59.2	−0.5	−0.98	−0.03	**4.35E−02**	**36.6**	0.126489	−0.94	0.52
	Creatinine	HMDB0000562	7.0	1.26E−01	2.49E−02	0.00E+00	1.68E−01	2.31E−01	−1.5	2.04E−02	−0.48	0.65	**4.16E−02**	**41.7**	**−0.5**	−0.92	−0.0027	**2.24E−02**	**84.5**	−0.4673	−1.03	0.06
	Phenylalanine	HMDB0000159	5.3	**1.66E−02**	**7.92E−02**	**2.66E−02**	**2.47E−01**	4.85E−01	67.9	−3.72E−01	−0.76	0.31	**1.64E−03**	**197.9**	**−0.8**	−1.2	−0.54	**3.29E−03**	**127.1**	−0.4175	−1.08	0.1
	Ornithine	HMDB0000214	37.3	1.21E−01	4.75E−02	0.00E+00	2.31E−01	4.18E−01	18.8	−9.58E−02	−0.62	0.51	**3.49E−02**	**187.0**	**−0.6**	−0.93	−0.1	**2.01E−02**	**160.3**	−0.48736	−0.92	0.0051
	Lysine	HMDB0000182	27.9	**2.77E−02**	**1.41E−02**	**0.00E+00**	**1.42E−01**	1.90E−01	86.8	−2.53E−01	−0.65	0.44	**8.99E−03**	**266.1**	**−0.4**	−0.76	−0.02	**2.84E−03**	**109.4**	−0.26582	−0.68	0.34
	Aspartic acid	HMDB0000191	5.4	5.20E−02	1.08E−01	3.68E−02	2.60E−01	2.27E−01	56.0	−5.22E−01	−1.06	0.01	**3.77E−03**	**196.9**	**−0.8**	−1.13	−0.52	**4.13E−02**	**103.5**	−0.56097	−0.98	−0.09
Beta hydroxy acids and derivatives	3-Hydroxybutyric acid	HMDB0000011	7.4	**2.43E−04**	**2.43E−01**	**1.57E−01**	**4.44E−01**	1.25E−01	−17.5	2.70E−01	−0.28	0.79	**6.32E−05**	**143.9**	**−1.0**	−1.42	−0.7	**5.36E−06**	**213.4**	−1.18135	−1.61	−0.85
Carbohydrates and carbohydrate conjugates	Erythritol	HMDB0002994	15.0	1.36E−01	1.89E−03	0.00E+00	1.11E−01	3.94E−01	−13.6	1.64E−01	−0.47	0.62	**2.47E−02**	**−31.3**	**0.4**	−0.12	0.78	**4.84E−02**	**−23.8**	0.265103	−0.28	0.83
	Threitol	HMDB0004136	7.1	8.84E−01	6.50E−02	0.00E+00	2.81E−01	9.76E−01	−53.8	3.85E−01	−0.09	0.62	5.20E−01	431.0	−0.2	−0.49	0.54	4.58E−01	965.3	−0.26002	−0.69	0.07
	Fucose	HMDB0000174	8.3	5.26E−02	4.51E−02	0.00E+00	2.61E−01	3.92E−01	−29.9	2.60E−01	−0.33	0.69	**1.44E−02**	**55.2**	**−0.4**	−0.96	0.1	**7.73E−03**	**146.2**	−0.72128	−1.16	−0.29
	Alpha-D-glucosamine phosphate	HMDB01109	26.3	**2.88E−03**	**1.85E−02**	**0.00E+00**	**1.40E−01**	3.09E−01	210.2	−3.11E−01	−0.66	0.25	**2.59E−04**	**−87.2**	**0.4**	0.32	1.37	**3.70E−04**	**−96.8**	0.468877	0.35	0.86
	Sorbitol	HMDB0000247	19.4	5.36E−02	3.16E−02	0.00E+00	2.61E−01	2.05E−01	−4.4	1.20E−02	−0.46	0.52	**4.70E−03**	**180.0**	**−0.2**	−0.49	0.53	5.58E−02	110.1	−0.21099	−0.48	0.57
Dicarboxylic acids and derivatives	Pentanedioic acid	HMDB0000661	5.0	2.53E−01	0.00E+00	0.00E+00	1.15E−01	1.50E−01	−35.7	3.45E−01	−0.19	0.79	1.60E−01	−1.9	0.0	−0.54	0.52	**4.53E−02**	**86.4**	−0.41386	−1.03	0.09
Fatty acids and conjugates	4-hydroxybutanoic acid	HMDB0000549	12.5	1.52E−01	0.00E+00	0.00E+00	1.73E−01	7.70E−02	−1.6	2.00E−02	−0.46	0.86	1.70E−01	31.0	−0.3	−0.89	0.17	**1.94E−02**	**73.0**	−0.28348	−1.12	0.23
Non-metal pyrophosphates	Pyrophosphate	HMDB0000250	10.6	5.36E−02	1.13E−01	1.81E−02	3.02E−01	3.23E−01	18.7	−2.49E−01	−0.79	0.3	**6.20E−03**	**100.9**	**−0.8**	−1.27	−0.4	**2.87E−02**	**78.0**	−0.66446	−1.15	−0.18
Pyrimidines and pyrimidine derivatives	Thymine	HMDB0000262	5.8	1.20E−01	0.00E+00	0.00E+00	2.18E−01	1.86E−01	38.5	−1.98E−01	−0.61	0.43	**1.23E−02**	**62.3**	**−0.5**	−1.03	0.05	1.13E−01	11.6	−0.12526	−0.99	0.33
Sulfinic acids	Hypotaurine	HMDB0000965	6.3	1.84E−01	0.00E+00	0.00E+00	1.41E−01	3.97E−01	5.2	−5.56E−02	−0.65	0.51	**3.27E−02**	**41.0**	**−0.4**	−0.95	0.13	6.09E−02	43.4	−0.34222	−0.92	0.2
Ureas	Urea	HMDB0000294	5.6	**1.45E−03**	**1.68E−01**	**7.43E−02**	**3.55E−01**	7.22E−02	9.1	−2.34E−01	−0.78	0.29	**8.91E−04**	**−34.5**	**0.9**	0.41	1.55	**2.19E−05**	**−41.9**	1.173124	0.7	1.86

**Fig. 1:**
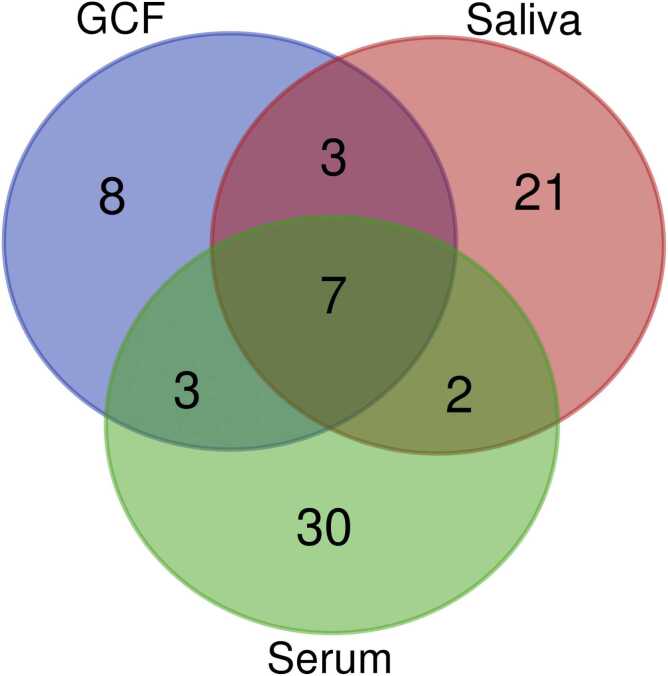
Venn diagram illustrating significantly altered metabolites (p < 0.05) identified in GCF, saliva, and serum.

Based on the data presented in Table S4 and the Venn diagram, 17 metabolites were found to be statistically significant in GCF samples across all comparisons (Fig. 1 and Table S2). Among them, 3-hydroxybutyric acid (3-HBA) was common to all comparisons. Myo-inositol and 2-monostearin were unique to the comparison between the ALMS+BBS group and Group III, while 14 metabolites were shared between the comparisons ALMS+BBS group and Group III and between the ALMS+BBS group and Group IV. In saliva samples, no significant differences in metabolite levels were observed between Ctrl and those with OB. Sorbitol, hypotaurine, and thymine were statistically significant only in the comparison between groups ALMS+BBS and Group IV. Meanwhile, threitol, GABA, proline, pentadienoic acid, and 4-hydroxybutanoic acid were significant in the comparison between the ALMS+BBS group and Group III (Table 2).

Among the 34 metabolites that were statistically significant in the serum samples, five metabolites (urea, ethanolamine, isoleucine, threonine, and ornithine) were unique to the comparison between the ALMS+BBS group and Group III, while six metabolites (alpha-tocopherol, glycine, pyruvic acid, alpha-ketoglutaric acid, fumaric acid, and sorbose) were unique to the comparison between groups the ALMS+BBS group and Group IV. Additionally, eleven metabolites (palmitic acid, linoleic acid, glycerol 1-phosphate, stearic acid, trans-13-octadecenoic acid, 3-HBA, methionine, 2-hydroxybutyric acid, acetoacetate, palmitoleic acid, and oleic acid) were common to both comparisons (Table S5).

**Fig. 2:**
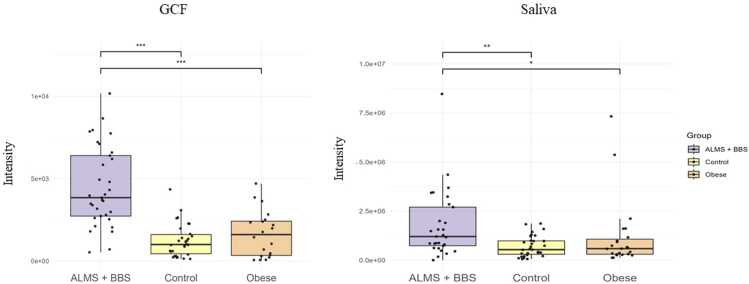
Boxplots comparing levels of phenylalanine in GCF GCF and saliva in ALMS+BBS patients, the comparative group (patients with simple obesity), and the healthy controls.

**Fig. 3:**
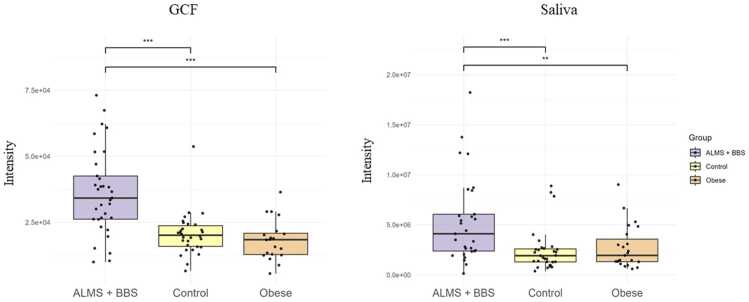
Boxplots comparing levels of alanine in GCF and saliva in ALMS+BBS patients, the comparative group (patients with simple obesity), and the healthy controls.

**Fig. 4:**
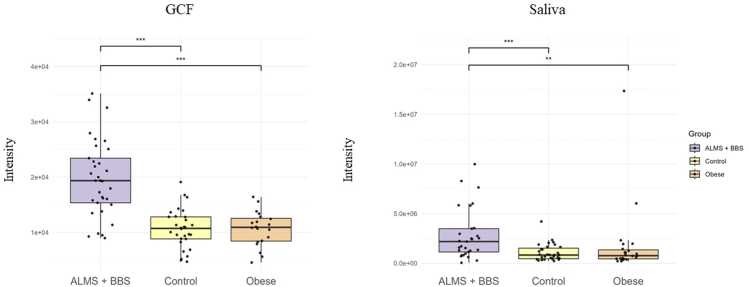
Boxplots comparing levels of valine in GCF and saliva in ALMS+BBS patients, the comparative group (patients with simple obesity), and the healthy controls.

**Fig. 5:**
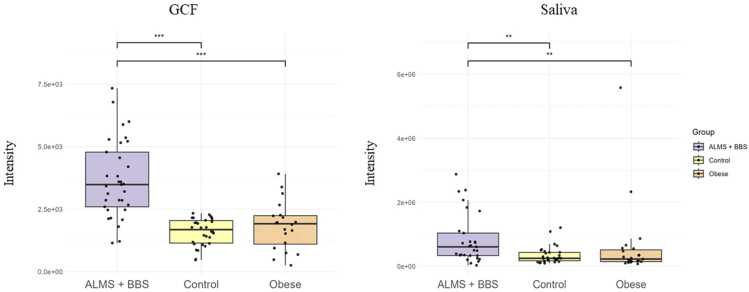
Boxplots comparing levels of isoleucine in GCF and saliva in ALMS+BBS patients, the comparative group (patients with simple obesity)**,** and the healthy controls.

In particular, an increase in the levels of selected metabolites was observed in both the GCF and saliva samples. Fig. 2, Fig. 3, Fig. 4, Fig. 5 present changes in phenylalanine, alanine, valine and isoleucine levels in the studied groups. Comparing the study group with Ctrl, the percentage changes in phenylalanine, alanine, valine, and isoleucine levels in saliva and GCF samples were 118 %/106 %, 207 %/175 %, 188 %/135 %, and 198 %/127 %, respectively. When compared with OB group, these changes were 106 %/97 %, 175 %/85 %, 135 %/101 %, and 127 %/172 %, respectively. Considering all statistically significant metabolites across all matrices, seven metabolites (valine, 3-HBA, alanine, threonine, urea, isoleucine, and phenylalanine) were consistently significant across all sample types, as shown in the Venn diagram (Fig. 1 and Table S2).

**Fig. 6:**
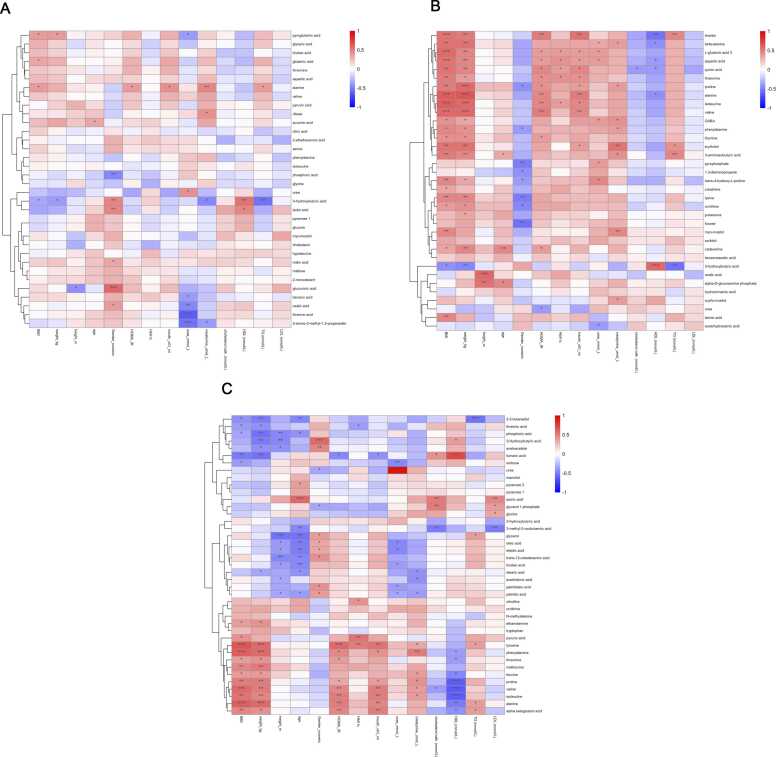
Heatmap of Spearman’s rank correlation analysis between differential metabolites and clinical data in A) GCF, B) saliva, and C) serum. Blue indicates a negative correlation; red indicates a positive correlation. Significant correlation regions are marked with stars (*p < 0.05, **p < 0.01).

Furthermore, in both the saliva and serum samples, positive correlations were observed between several amino acids—including aromatic amino acids (AAAs), branched-chain amino acids (BCAAs), proline, threonine and lysine and ornithine—and HOMA-IR, HbA1c, and insulin levels (Fig. 6 B, C). In the saliva samples, HDL levels were strongly positively correlated with 3-HBA (*p* = 5.268914 × 10–4) and negatively correlated with alanine (*p* = 1.44357664 × 10–2), beta-alanine (*p* = 2.44948043 × 10–2), and aspartic acid (*p* = 4.17092046 × 10–2). In the serum samples, HDL levels were negatively correlated with the amino acids phenylalanine, threonine, leucine, proline, valine, isoleucine and alanine. Finally, strong positive correlations were confirmed between amino acid-related metabolites and both BMI and weight across both matrices (saliva and serum) (Fig. 6 B, C).

A correlation analysis was also conducted between the statistically significant GCF metabolites and clinical parameters. In the GCF samples from the study group, alanine showed strong positive correlations with TG (*p* = 3.679479 × 10–2), creatinine (*p* = 8.853197 × 10–3), and insulin levels (*p* = 2.00218 × 10–2), as well as with HOMA-IR (*p* = 2.821387 × 10–2). Threonic acid (*p* = 1.59703 × 10–4) and oxalic acid (*p* = 9.8263933 × 10–3) exhibited strong negative correlations with urea concentration. 3-HBA demonstrated a strong negative correlation with TG level (*p* = 1.542835 × 10–3) and a strong positive correlation with HDL level (*p* = 1.305216 × 10–3). Finally, lactic acid also showed a positive correlation with HDL level (*p* = 1.17167719 × 10–2) (Fig. 6A).

**Fig. 7:**
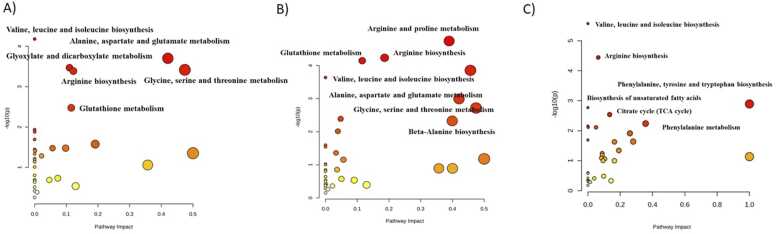
The results of biochemical pathways analysis for metabolites detected by GC–MS in A) GCF, B) saliva and C) serum samples. Each circle represents a metabolic pathway. The x-axis shows the pathway impact, indicating the relative importance of the pathway based on topology analysis, while the y-axis (−log10(p)) reflects the statistical significance of pathway enrichment. The size of the circles corresponds to the magnitude of pathway impact, and the color scale (from yellow to red) indicates increasing levels of statistical significance, with red representing the most significantly affected pathways.

Pathway analysis based on GC–MS data in MetaboAnalyst 5.0 revealed significant metabolic disturbances in GCF, saliva, and serum between ALMS/BBS patients and controls (Fig. 7). The analysis of statistically significant metabolites showed both overlapping and matrix-specific metabolic disturbances. Common alterations were observed across GCF, saliva, and serum, including disruptions in valine, leucine and isoleucine, as well as arginine biosynthesis. In addition, the metabolism of glutathione, glycine, serine and threonine, as well as the metabolism of alanine, aspartate, and glutamate were affected in both GCF and saliva, but not in serum (Fig. 7 and Table S3).

## 4 Discussion

This study provides comprehensive metabolomic profiling of patients with ALMS and BBS syndromes, including heterozygous carriers of these disorders. Importantly, statistical analyses revealed no significant differences in metabolomic matrices between homozygous and heterozygous patients, nor between carriers of causative variants in the *ALMS1* and *BBS* genes, allowing us to combine them into a single study group. These results were further supported by effect size analyses, which showed only small or moderate differences between homozygous and heterozygous individuals. To the best of our knowledge, this is the first study to use untargeted GC-MS to evaluate the metabolomic signatures across three matrices - GCF, saliva, and serum - in these patients and to compare the results with healthy controls and individuals with simple obesity. Although different biofluids were used, we were able to differentiate consistent patterns, particularly a positive correlation between amino acid-related metabolites and BMI/weight across all three matrices, reinforcing the systemic nature of metabolic disruption in both syndromes.

We identified a consistent panel of seven significantly altered metabolites across all matrices - valine, isoleucine, alanine, threonine, phenylalanine, urea, and 3-HBA - strongly implicating amino acid dysregulation and altered energy metabolism in these syndromes. The increased levels of valine and isoleucine (BCAAs) and phenylalanine (AAA) in the GCF and saliva samples are consistent with previous reports linking these metabolites with insulin resistance and obesity [33], [34].

Furthermore, the increased levels of phenylalanine, an aromatic amino acid, could indicate broader disruptions in AAA metabolism, potentially linked to oxidative stress and neurochemical imbalance [35], [36]. In these genetic syndromes, where cognitive and neurological symptoms are more variable [16], phenylalanine may differentiate patients with neurodevelopmental problems.

The correlation analyses revealed positive associations between BCAAs and hyperinsulinemia and insulin resistance. This finding is consistent with the literature, which has previously shown that BCAAs activate the mTOR pathway, and disrupt insulin signaling [37], [38]. Altered levels of BCAAs and the phenylalanine (AAA) may underlie the observed metabolic disturbances in both ALMS and BBS syndromes [39]. Elevated BCAAs, commonly associated with insulin resistance and obesity, may reflect impaired mitochondrial function and defective amino acid catabolism, contributing to the development of type 2 diabetes in both disorders [34], [37], [40]. Notably, Newgard et al. suggested a causal rather than consequential role of BCAA elevation in the development of insulin resistance [33]. Additionally, a recent serum-based study by Zhang et al. [41] in syndromic obesity reported elevated BCAA/AAA levels in BBS patients with insulin resistance, partially supporting our results but lacking multi-fluid validation. Moreover, McCafferty et al. found various changes in amino acids in motile cilia, linking these discrepancies to protein complexes involved in ciliopathies and providing a molecular basis for further research into changes in amino acid metabolism in these diseases [42].

Interestingly, we confirmed the elevation of 3-HBA, a ketone body with protective metabolic properties, in all studied matrices when comparing the study group with the OB and Ctrl groups. The increase in 3-HBA levels was positively correlated with HDL and negatively with triglycerides in GCF, indicating a possible compensatory metabolic response to impaired glucose utilization or insulin resistance. Prior studies have noted 3-HBA’s protective roles in modulating oxidative stress, mitochondrial function, and lipid metabolism [43]. This raises the question of whether 3-HBA can serve as a protective biomarker reflecting a healthy metabolic balance. However, given the cross-sectional nature of the study, this observation may provide an interesting starting point for further longitudinal studies to confirm our hypothesis.

In addition, the results also indicate a link between selected metabolites - in particular, BCAA and ketone bodies - and disorders such as overweight/obesity or hyperglycemia observed in heterozygous carriers of *ALMS1* and *BBS* gene variants. This highlights the importance of close clinical monitoring of these patients. Previous studies have suggested a possible “dose-dependent” metabolic liability in heterozygous individuals, indicating that metabolic alterations may precede or accompany clinical manifestations, providing a potential window for early diagnosis and intervention [44], [45]. Furthermore, the metabolomic profiling performed here may help to differentiate syndromic obesity from simple obesity, enabling more personalized clinical management for these distinct disorders.

Furthermore, lipid-related metabolic pathways were also notably impaired in our patients, especially in serum, including unsaturated fatty acid biosynthesis, glycerolipid metabolism, and short-chain fatty acid catabolism. These findings align with clinical features such as dyslipidemia and ectopic fat accumulation [46], [47].

Pathway enrichment analysis revealed both shared and matrix-specific metabolic disruptions in ALMS and BBS patients. The biosynthesis of branched-chain amino acids (BCAAs) and arginine was consistently altered across all matrices, indicating a consistent metabolic disturbance compared with healthy controls. In serum, pathways related to energy and fatty acid metabolism, including the TCA cycle and biosynthesis of unsaturated fatty acids, were primarily affected, reflecting systemic metabolic changes. In contrast, GCF and saliva showed the greatest alterations in amino acid and glutathione metabolism, suggesting that local oral metabolic processes are also impacted. Overall, these findings indicate that while some metabolic disruptions are systemic, others are matrix-specific, highlighting the distinct biological roles of serum and oral fluids and emphasising the characteristic metabolic signature of ALMS and BBS syndromes.

Thus, the analysis of metabolomic signatures in all three matrices was complementary, revealing both similarities and differences in various biological fluids, which may lead to the differentiated use of individual matrices. Our findings appear to confirm the hypothesis of a common metabolic phenotype of these genetic disorders, characterized by abnormalities in amino acid metabolism, lipid pathways, and oxidative stress, distinguishing affected patients from those with simple obesity. Importantly, the detection of altered metabolite profiles in saliva and GCF - non-invasive and easily accessible biofluids - highlights their potential utility in metabolic monitoring. This is particularly relevant for pediatric and medically fragile populations, where serum sampling may be challenging. Furthermore, a comparative analysis between matrices showed that serum exhibited the most consistent and significant metabolic changes, best reflecting systemic changes associated with ALMS and BBS. However, several metabolites were detectable exclusively or showed matrix-specific trends in saliva (21 unique metabolites) and GCF (8 unique metabolites), highlighting their complementary role as non-invasive biological fluids for further translational studies.

Although our findings provide new insights, the study’s limitations include the small cohort size due to the rarity of ALMS and BBS, and the cross-sectional design, which precludes causal inference. Unfortunately, we did not have detailed clinical data for patients with OB and for Ctrl, which made it impossible to assess the correlation with metabolites in these groups. Further studies with larger cohorts and longitudinal designs are needed to determine causality and assess the clinical utility of these metabolic markers. Nevertheless, our data are consistent with previous studies on rare monogenic diabetes and obesity syndromes, and extend these findings by adding multi-fluid, non-invasive metabolomic profiling.

## 5 Conclusions

In conclusion, this study provides a comprehensive, multi-fluid, untargeted metabolomic analysis in relation to causative variants in *ALMS1* and *BBS* genes, identifying common metabolic abnormalities. We found key changes in amino acid metabolism, oxidative stress pathways, and lipid metabolism in patients and heterozygous variant carriers, associated with early obesity, insulin resistance, and hyperglycemia. These findings underscore the potential of metabolomics - particularly when using non-invasive oral fluids - to detect early metabolic disorders, positioning selected metabolites as promising biomarkers. This offers opportunities for early intervention and personalized treatment for patients with disease-associated variants in the *ALMS1* and *BBS* genes.

## CRediT authorship contribution statement

**Sebastian Skoczylas:** Methodology. **Krzysztof Sołowiej:** Methodology. **Sandra Chmielewska:** Methodology. **Julia Grzybowska-Adamowicz:** Investigation. **Agnieszka Zmysłowska:** Data curation. **Michał Ciborowski:** Writing – review & editing. **Ewa Zmysłowska-Polakowska:** Writing – original draft, Conceptualization. **Patrycja Mojsak:** Methodology, Investigation. **Aleksandra Palatynska-Ulatowska:** Investigation. **Monika Lukomska-Szymanska:** Investigation. **Adam Kretowski:** Methodology.

## Funding

Ministry of Education and Science within the project “Excellence Initiative - Research University”, Medical University of Bialystok (B.SUB.25.522).

## Declaration of Competing Interests

All authors read and approved the final version of this manuscript and declare that they have no competing financial interests in relation to this work. We confirm that neither the manuscript nor any parts of its content are currently under consideration or published in another journal.

## Appendix A Supplementary material

Supplementary material

Supplementary material **Figure S1.** PCA analysis showing QC sample distribution (green dots) in A) GCF (data were logarithmized, univariate scaling was applied, R^2^ = 0.472), B) saliva (data were logarithmized, Pareto scaling was applied, R^2^ = 0.784), and C) serum GCF (data were logarithmized, univariate scaling was applied, R^2^ = 0.541)

Supplementary material **Figure S2**. Partial least squares discriminant analysis (PLS-DA) score plots of the serum fingerprints of ALMS/BBS patients (red dots), participants with obesity (blue dots) and the control group (yellow dots). A) GCF: R2 = 0.608, Q2 = 0.464 (*p* = 1.31 ×10 ^−3^), B) saliva, R2 = 0.473, Q2 = 0.247 (*p* = 4.1 ×10 ^−2^), C) serum R2 = 0.453, Q2 = 0.343 (*p* = 3.74 ×10 ^−5^)

Supplementary material **Figure S3.** Effect sizes (rank-biserial correlations) for metabolites differentiating ALMS+BBS homozygous and heterozygous groups in GCF

Supplementary material **Figure S4.** Effect sizes (rank-biserial correlations) for metabolites differentiating ALMS+BBS homozygous and heterozygous groups in saliva

Supplementary material **Figure S5.** Effect sizes (rank-biserial correlations) for metabolites differentiating ALMS+BBS homozygous and heterozygous groups in serum
