# Supplementary material for: Multi-matrix metabolomics in rare monogenic diabetes syndromes: Analysis of oral fluids and serum in carriers of pathogenic variants in the ALMS1/BBS genes
Source: Comput Struct Biotechnol J. 2025 Oct 22;27:4880–9. doi: 10.1016/j.csbj.2025.10.040 (PMC12648480; doi:10.1016/j.csbj.2025.10.040)
Supplement: Supplementary file 1 — Supplementary material [file mmc1.docx]

| **No.** | **Metabolite** | | **Sample type** | **RT** | **TI** | **QI 1** | **QII 2** | **RI** | **HMDB** | **Class** | **Subclass** |
| --- | --- | --- | --- | --- | --- | --- | --- | --- | --- | --- | --- |
| **1 TMS** | Boric acid |  | serum. saliva | 5.3 | 221 | 236 | 133 | 993 | HMDB0034769 | Metalloid organides | Metalloid oxides |
| **2** | 2-3 butanediol | 2-3 butanediol 1 TMS | serum, saliva | 5.9 | 117 | 73 | 147 | 703 | HMDB0003156 | Organooxygen compounds | Alcohols and polyols |
|  |  | 2-3 butanediol 2 TMS | serum, saliva | 6.1 | 117 | 73 | 147 | 712 |  |  |  |
| **3** | Pyruvic acid |  | serum, saliva, GCF | 6.2 | 174 | 89 | 59 | 720 | HMDB0000243 | Keto acids and derivatives | Alpha-keto acids and derivatives |
| **4** | Lactic acid |  | serum, saliva, GCF | 6.3 | 117 | 191 | 133 | 732 | HMDB0000190 | Hydroxy acids and derivatives | Alpha hydroxy acids and derivatives |
| **5** | N-ethylglycine |  | serum, saliva | 6.4 | 58 | 75 | 73 | 706 | HMDB0041945 | Carboxylic acids and derivatives | Amino acids, peptides, and analogues |
| **6** | Carbamic acid |  | serum | 6.4 | 103 | 118 | 100 | 699 | HMDB0003551 | Organic carbonic acids and derivatives | nd |
| **7** | Glycolic acid |  | serum, saliva | 6.6 | 66 | 177 | 133 | 747 | HMDB0000115 | Hydroxy acids and derivatives | Alpha hydroxy acids and derivatives |
| **8** | N,N-dimethylglycine |  | serum, saliva | 6.7 | 59 | 160 | 175 | 697 | HMDB0000092 | Carboxylic acids and derivatives | Amino acids, peptides, and analogues |
| **9** | Valine | Valine 1 TMS | serum, GCF | 6.8 | 72 | 55 | 75 | 763 | HMDB0000883 | Carboxylic acids and derivatives | Amino acids, peptides, and analogues |
|  |  | Valine 2 TMS | serum, GCF | 8.7 | 144 | 218 | 73 | 898 |  |  |  |
| **10** | Alanine | Alanine 1 TMS | serum, saliva, GCF | 7.0 | 116 | 73 | 147 | 774 | HMDB0000161 | Carboxylic acids and derivatives | Amino acids, peptides, and analogues |
|  |  | Alanine 2 TMS | serum, saliva, GCF | 10.7 | 188 | 100 | 262 | 1024 |  |  |  |
| **11** | 3-methyl-2-oxobutanoic acid | 3-methyl-2-oxobutanoic acid 1 TMS | serum | 7.1 | 89 | 202 | 186 | 806 | HMDB0030027 | Fatty Acyls | Fatty acid esters |
|  |  | 3-methyl-2-oxobutanoic acid 2 TMS | serum | 7.4 | 89 | 202 | 186 | 806 |  |  |  |
| **12** | Acetohydroxamic acid |  | serum, saliva | 7.2 | 133 | 119 | 249 | 794 | HMDB0014691 | Carboxylic acids and derivatives | Carboxylic acid derivatives |
| **13** | Acetoacetate | Acetoacetate 1 TMS | serum | 7.2 | 89 | 59 | 186 | 785 | HMDB0304256 | Keto acids and derivatives | Short-chain keto acids and derivatives |
|  |  | Acetoacetate 2 TMS | serum | 7.4 | 73 | 89 | 202 | 807 |  |  |  |
| **14** | Glycine | Glycine 1 TMS | serum, saliva, GCF | 7.3 | 102 | 147 | 73 | 985 | HMDB0000123 | Carboxylic acids and derivatives | Amino acids, peptides, and analogues |
|  |  | Glycine 3 TMS | serum, saliva, GCF | 10.0 | 174 | 248 | 147 | - |  | Carboxylic acids and derivatives | Amino acids, peptides, and analogues |
| **15** | 2-hydroxybutyric acid |  | serum, saliva | 7.3 | 131 | 75 | 205 | 800 | HMDB0000011 | Hydroxy acids and derivatives | Beta hydroxy acids and derivatives |
| **16** | Lactamide |  | saliva | 7.5 | 117 | 100 | 89 | 808 | HMDB0253942 | Organooxygen compounds | Alcohols and polyols |
| **17** | Oxalic acid |  | serum, saliva, GCF | 7.6 | 190 | 147 | 190 | 806 | HMDB0002329 | Carboxylic acids and derivatives | Dicarboxylic acids and derivatives |
| **18** | Hydracrylic acid |  | serum, saliva | 7.6 | 219 | 177 | 41 | - | HMDB0000700 | Hydroxy acids and derivatives | Beta hydroxy acids and derivatives |
| **19** | p-cresol |  | serum, saliva | 7.8 | 165 | 180 | 91 | 828 | HMDB0001858 | Phenols | Cresols |
| **20** | Leucine | Leucine 1 TMS | serum | 7.8 | 86 | 75 | 73 | 832 | HMDB0000687 | Carboxylic acids and derivatives | Amino acids, peptides, and analogues |
|  |  | Leucine 2 TMS | serum | 9.5 | 158 | 73 | 232 | 946 |  |  |  |
| **21** | 3-Hydroxybutyric acid |  | serum, saliva, GCF | 7.8 | 57 | 59 | 87 | - | HMDB0000011 | Hydroxy acids and derivatives | Beta hydroxy acids and derivatives |
| **22** | 2-Hydroxy-3-methylbutyric acid |  | serum, saliva | 7.9 | 145 | 45 | 59 | - | HMDB0000407 | Fatty Acyls | Fatty acids and conjugates |
| **23** | 2-Ethylhexanoic acid |  | GCF | 7.9 | 201 | 160 | 75 | - | HMDB0031230 | Fatty Acyls | Fatty acids and conjugates |
| **24** | N-methylalanine |  | serum | 8.0 | 130 | 77 | 73 | - | HMDB0094692 | Carboxylic acids and derivatives | Amino acids, peptides, and analogues |
| **25** | 2-ketoisocaproic acid | 2-ketoisocaproic acid 1 TMS | serum | 8.1 | 189 | 89 | 189 | 851 | HMDB0000695 | Keto acids and derivatives | Short-chain keto acids and derivatives |
|  |  | 2-ketoisocaproic acid 2 TMS | serum | 8.6 | 200 | 216 | 189 | 886 |  |  |  |
| **26** | Proline | Proline 1 TMS | serum, saliva | 8.1 | 70 | 75 | 68 | 851 | HMDB0000162 | Carboxylic acids and derivatives | Amino acids, peptides, and analogues |
|  |  | Proline 2 TMS | serum, saliva | 9.9 | 142 | 216 | 143 | 977 |  |  |  |
| **27** | Isoleucine | Isoleucine 1 TMS | serum, saliva, GCF | 8.1 | 86 | 69 | 73 | 853 | HMDB0000172 | Carboxylic acids and derivatives | Amino acids, peptides, and analogues |
|  |  | Isoleucine 2 TMS | serum, saliva, GCF | 9.8 | 158 | 218 | 73 | 965 |  |  |  |
| **28** | Mimosine |  | serum | 8.4 | 188 | 216 | 172 | 873 | HMDB0015188 | Carboxylic acids and derivatives | Amino acids, peptides, and analogues |
| **29** | β-hydroxyisovalerate |  | serum | 8.6 | 131 | 247 | 73 | 888 | HMDB0000754 | Fatty Acyls | Fatty acids and conjugates |
| **30** | 4-hydroxybutanoic acid |  | saliva | 9.0 | 117 | 233 | 75 | - | HMDB0000710 | Fatty Acyls | Fatty acids and conjugates |
| **31** | Urea |  | serum, GCF | 9.1 | 189 | 66 | 147 | 924 | HMDB0000294 | Organic carbonic acids and derivatives | Ureas |
| **32** | Benzoic acid |  | serum, GCF | 9.2 | 179 | 105 | 135 | 935 | HMDB0001870 | Benzene and substituted derivatives | Benzoic acids and derivatives |
| **33** | Ethanolamine |  | serum, saliva | 9.4 | 174 | 73 | 147 | 941 | HMDB0000149 | Organonitrogen compounds | Amines |
| **34** | Phosphoric acid |  | serum, saliva, GCF | 9.5 | 299 | 314 | 73 | 948 | HMDB0001429 | Non-metal oxoanionic compounds | Non-metal phosphates |
| **35** | Glycerol |  | serum | 9.5 | 205 | 117 | 103 | 950 | HMDB0000131 | Organooxygen compounds | Carbohydrates and carbohydrate conjugates |
| **36** | 2-amino-2-methyl-1,3-propanediol |  | saliva, GCF | 9.7 | 74 | 56 | 28 | - | HMDB0244975 | Organonitrogen compounds | Amines |
| **37** | Benzeneacetic acid |  | saliva | 9.9 | 91 | 136 | 65 | - | HMDB0259453 | Phenol ethers | nd |
| **38** | Succinic acid |  | serum, GCF | 10.1 | 75 | 55 | 129 | 989 | HMDB0000254 | Carboxylic acids and derivatives | Dicarboxylic acids and derivatives |
| **39** | Fumaric acid |  | serum, saliva | 10.2 | 245 | 147 | 73 | 1003 | HMDB0000134 | Carboxylic acids and derivatives | Dicarboxylic acids and derivatives |
| **40** | Glyceric acid |  | serum, saliva, GCF | 10.3 | 189 | 292 | 133 | 986 | HMDB0000139 | Organooxygen compounds | Carbohydrates and carbohydrate conjugates |
| **41** | Glyoxylic acid |  | serum, saliva | 10.4 | 184 | 134 | 285 | 992 | HMDB0000119 | Carboxylic acids and derivatives | Carboxylic acids |
| **42** | Citraconic acid |  | saliva | 10.4 | 241 | 99 | 255 | 991 | HMDB0000634 | Fatty Acyls | Fatty acids and conjugates |
| **43** | Itaconic acid |  | saliva | 10.6 | 259 | 230 | 75 | 995 | HMDB0002092 | Fatty Acyls | Fatty acids and conjugates |
| **44** | Tartronic acid |  | serum | 10.6 | 102 | 292 | 133 | 1053 | HMDB0035227 | Carboxylic acids and derivatives | Dicarboxylic acids and derivatives |
| **45** | Serine | Serine 1 TMS | serum, GCF | 9.3 | 116 | 132 | 75 | 928 | HMDB0000187 | Carboxylic acids and derivatives | Amino acids, peptides, and analogues |
|  |  | Serine 2 TMS | serum, GCF | 10.7 | 204 | 218 | 100 | 1023 |  |  |  |
| **46** | Pelargonic acid (nonanoic acid) |  | serum, saliva | 10.8 | 215 | 215 | 215 | 1019 | HMDB0000847 | Fatty Acyls | Fatty acids and conjugates |
| **47** | Threonine | Threonine 1 TMS | serum, GCF | 9.8 | 117 | 130 | 219 | 968 | HMDB0000167 | Carboxylic acids and derivatives | Amino acids, peptides, and analogues |
|  |  | Threonine 2 TMS | serum, GCF | 11.1 | 218 | 117 | 291 | 1048 |  |  |  |
| **48** | Thymine |  | saliva | 11.3 | 255 | 270 | 113 | 1059 | HMDB0000262 | Diazines | Pyrimidines and pyrimidine derivatives |
| **49** | Methionine | Methionine 1 TMS | serum | 11.5 | 104 | 61 | 56 | 1079 | HMDB0000696 | Carboxylic acids and derivatives | Amino acids, peptides, and analogues |
|  |  | Methionine 2 TMS | serum | 12.9 | 176 | 128 | 61 | 1195 |  |  |  |
| **50** | Hydrocinnamic acid |  | saliva | 11.6 | 104 | 75 | 207 | 1085 | HMDB0000764 |  |  |
| **51** | Aspartic acid | Aspartic acid 1 TMS | serum, saliva, GCF | 11.6 | 160 | 130 | 117 | 1094 | HMDB0000191 | Carboxylic acids and derivatives | Amino acids, peptides, and analogues |
|  |  | Aspartic acid 2 TMS | serum, saliva, GCF | 12.9 | 232 | 100 | 218 | 1196 |  |  |  |
| **52** | β-alanine |  | serum, saliva | 11.7 | 174 | 248 | 290 | 1097 | HMDB0000056 | Carboxylic acids and derivatives | Amino acids, peptides, and analogues |
| **53** | 3-aminoisobutyric acid |  | saliva | 12.1 | 174 | 248 | 304 | 1132 | HMDB0003911 | Carboxylic acids and derivatives | Amino acids, peptides, and analogues |
| **54** | Capric acid |  | serum | 12.1 | 229 | 117 | 129 | 1128 | HMDB0000511 | Fatty Acyls | Fatty acids and conjugates |
| **55** | Aminomalonic acid |  | saliva | 12.2 | 218 | 320 | 174 | - | HMDB0001147 | Carboxylic acids and derivatives | Amino acids, peptides, and analogues |
| **56** | trans-4-hydroxy-L-proline | trans-4-hydroxy-L-proline 1 TMS | serum | 12.3 | 158 | 68 | 260 | 1146 | HMDB0000725 | Carboxylic acids and derivatives | Amino acids, peptides, and analogues |
|  |  | trans-4-hydroxy-L-proline 2 TMS | serum | 13.0 | 230 | 140 | 73 | 1181 |  |  |  |
| **57** | Malic acid |  | serum, saliva, GCF | 12.5 | 233 | 133 | 245 | 1164 | HMDB0031518 | Hydroxy acids and derivatives | Beta hydroxy acids and derivatives |
| **58** | 3,4-dimethylbenzoic acid |  | saliva | 12.6 | 207 | 163 | 222 | - | HMDB0002237 | Benzene and substituted derivatives | Benzoic acids and derivatives |
| **59** | Erythritol |  | serum, saliva | 12.6 | 61 | 44 | 91 | - | HMDB0002994 | Organooxygen compounds | Carbohydrates and carbohydrate conjugates |
| **60** | Threitol |  | serum, saliva | 12.7 | 217 | 217 | 147 | 1177 | HMDB0004136 | Organooxygen compounds | Carbohydrates and carbohydrate conjugates |
| **62** | Pyroglutamic acid |  | GCF | 12.9 | 156 | 230 | 258 | 1196 | HMDB0000267 | Carboxylic acids and derivatives | Amino acids, peptides, and analogues |
| **63** | γ-aminobutyric acid (GABA) |  | saliva | 13.0 | 174 | 304 | 86 | 1186 | HMDB0000112 | Carboxylic acids and derivatives | Amino acids, peptides, and analogues |
| **64** | Glutamic acid | Glutamic acid 1 TMS | serum, saliva, GCF | 13.1 | 84 | 174 | 75 | 1298 | HMDB0000148 | Carboxylic acids and derivatives | Amino acids, peptides, and analogues |
|  |  | Glutamic acid 2 TMS | serum, saliva, GCF | 14.1 | 246 | 128 | 156 | 1287 |  |  |  |
| **65** | Guanidinoacetic acid |  | serum | 13.3 | 171 | 329 | 198 | 1236 | HMDB0000128 | nd | nd |
| **66** | Creatinine |  | serum, saliva | 13.3 | 115 | 100 | 73 | 1233 | HMDB0000562 | Carboxylic acids and derivatives | Amino acids, peptides, and analogues |
| **67** | Threonic acid |  | serum, GCF | 13.4 | 292 | 205 | 220 | 1235 | HMDB0000943 | Organooxygen compounds | Carbohydrates and carbohydrate conjugates |
| **68** | α-ketoglutaric acid |  | serum | 13.6 | 198 | 147 | 75 | 1250 | HMDB0000208 | Keto acids and derivatives | Gamma-keto acids and derivatives |
| **69** | Pentanedioic acid |  | saliva | 13.6 | 86 | 42 | 55 | 1231 | HMDB0000661 | Carboxylic acids and derivatives | Dicarboxylic acids and derivatives |
| **70** | 3-phenyllactic acid |  | saliva | 13.7 | 193 | 220 | 91 | 1248 | HMDB0000779 | Benzene and substituted derivatives | nd |
| **71** | Hypotaurine |  | serum, saliva, GCF | 13.9 | 188 | 100 | 174 | 1260 | HMDB0000965 | Sulfinic acids and derivatives | Sulfinic acids |
| **72** | Phenylalanine | Phenylalanine 1 TMS | serum, saliva, GCF | 13.3 | 120 | 146 | 73 | 1226 | HMDB0000159 | Carboxylic acids and derivatives | Amino acids, peptides, and analogues |
|  |  | Phenylalanine 2 TMS | serum, saliva, GCF | 14.2 | 218 | 192 | 73 | 1303 |  |  |  |
| **73** | 5-aminovaleric acid | 5-aminovaleric acid 1 TMS | saliva | 14.2 | 174 | 82 | 200 | 1293 | HMDB0003355 | Carboxylic acids and derivatives | Amino acids, peptides, and analogues |
|  |  | 5-aminovaleric acid 2 TMS | saliva | 14.8 | 174 | 200 | 86 | 1340 |  |  |  |
|  |  | 5-aminovaleric acid 3 TMS | saliva | 15.2 | 174 | 200 | 86 | 1377 |  |  |  |
| **74** | 1,3-diaminopropane |  | saliva | 14.4 | 174 | 160 | 201 | 1316 | HMDB0000002 | Organonitrogen compounds | Amines |
| **75** | Lauric acid |  | serum | 14.6 | 257 | 117 | 73 | 1329 | HMDB0000638 | Fatty Acyls | Fatty acids and conjugates |
| **76** | Pyrophosphate |  | saliva | 14.6 | 451 | 466 | 73 | 1346 | HMDB0304340 | Non-metal oxoanionic compounds | Non-metal pyrophosphates |
| **77** | Arabinose |  | serum | 14.6 | 103 | 217 | 307 | 1334 | HMDB0000646 | Organooxygen compounds | Carbohydrates and carbohydrate conjugates |
| **78** | Taurine |  | saliva | 14.7 | 326 | 188 | 100 | 1338 | HMDB0000251 | Organic sulfonic acids and derivatives | Organosulfonic acids and derivatives |
| **79** | Ribose |  | GCF | 14.8 | 103 | 217 | 307 | 1355 | HMDB0000283 | Organooxygen compounds | Carbohydrates and carbohydrate conjugates |
| **80** | Xylitol |  | serum | 14.8 | 103 | 205 | 307 | 1379 | HMDB0242149 | Organooxygen compounds | Carbohydrates and carbohydrate conjugates |
| **81** | Arabitol |  | serum | 15.3 | 217 | 103 | 205 | 1381 | HMDB0000568 | Organooxygen compounds | Carbohydrates and carbohydrate conjugates |
| **82** | Rhamnose |  | serum | 15.4 | 117 | 160 | 277 | 1398 | HMDB0000849 | Organooxygen compounds | Carbohydrates and carbohydrate conjugates |
| **83** | Fucose | Fucose 1 TMS | saliva | 15.4 | 117 | 160 | 277 | 1383 | HMDB0000174 | Organooxygen compounds | Carbohydrates and carbohydrate conjugates |
|  |  | Fucose 2 TMS | saliva | 15.5 | 117 | 160 | 277 | 1399 |  |  |  |
| **84** | Putrescine |  | saliva | 15.5 | 174 | 214 | 200 | 1392 | HMDB0001414 | Organonitrogen compounds | Amines |
| **85** | Dihydroxyacetone phosphate |  | saliva | 15.6 | 315 | 299 | 400 | 1402 | HMDB0001473 | Organooxygen compounds | Carbohydrates and carbohydrate conjugates |
| **86** | Citrulline |  | serum | 15.6 | 70 | 184 | 75 | 1404 | HMDB0000904 | Carboxylic acids and derivatives | Amino acids, peptides, and analogues |
| **87** | Orotic acid |  | saliva | 15.6 | 254 | 357 | 100 | 1400 | HMDB0000226 | Diazines | Pyrimidines and pyrimidine derivatives |
| **88** | Ornithine | Ornithine 1 TMS | serum, saliva | 15.7 | 142 | 70 | 102 | 1283 | HMDB0000214 | Carboxylic acids and derivatives | Amino acids, peptides, and analogues |
|  |  | Ornithine 2 TMS | serum, saliva | 16.4 | 142 | 174 | 200 | 1488 |  |  |  |
| **89** | Glycerol 1-phosphate |  | serum, saliva | 15.8 | 299 | 357 | 211 | 1428 | HMDB0000126 | Glycerophospholipids | Glycerophosphates |
| **90** | 3-(4-hydroxyphenyl)propionic acid |  | saliva | 15.8 | 179 | 192 | 310 | 1420 | HMDB0002199 | Phenylpropanoic acids | nd |
| **91** | O-phosphocolamine (Synonym: O-phosphorylethanolamine) |  | saliva | 16.0 | 299 | 174 | 188 | 1446 | HMDB0000224 | Organic phosphoric acids and derivatives | Phosphate esters |
| **92** | Isophthalic acid |  | saliva | 16.1 | 149 | 166 | 121 | - | HMDB0253671 | Benzene and substituted derivatives | nd |
| **93** | α-D-glucosamine phosphate |  | saliva | 16.3 | 217 | 189 | 232 | 1487 | HMDB0001109 | Organooxygen compounds | Carbohydrates and carbohydrate conjugates |
| **94** | 3-phosphoglyceric acid |  | saliva | 16.3 | 299 | 357 | 227 | 1474 | HMDB0000807 | Organooxygen compounds | Carbohydrates and carbohydrate conjugates |
| **95** | Hypoxanthine |  | serum | 16.3 | 265 | 280 | 73 | 1472 | HMDB0000157 | Imidazopyrimidines | Purines and purine derivatives |
| **96** | Citric acid |  | serum, saliva, GCF | 16.4 | 273 | 347 | 375 | 1494 | HMDB0000094 | Carboxylic acids and derivatives | Tricarboxylic acids and derivatives |
| **97** | Cadaverine |  | saliva | 16.7 | 174 | 102 | 128 | - | HMDB0002322 | Organonitrogen compounds | Amines |
| **98** | Dehydroascorbic acid |  | serum | 16.7 | 173 | 157 | 316 | 1511 | HMDB0001264 | Lactones | Gamma butyrolactones |
| **99** | Hippuric acid |  | serum | 16.8 | 105 | 206 | 77 | 1512 | HMDB0000714 | Benzene and substituted derivatives | Benzoic acids and derivatives |
| **100** | 1,5-anhydro-D-sorbitol |  | serum, saliva | 16.8 | 217 | 191 | 204 | 1522 | HMDB0244228 | Organooxygen compounds | Carbohydrates and carbohydrate conjugates |
| **101** | Lysine |  | saliva | 16.8 | 200 | 175 | 156 | 1527 | HMDB0000182 | Carboxylic acids and derivatives | Amino acids, peptides, and analogues |
| **102** | Quinic acid |  | serum | 16.9 | 345 | 255 | 204 | 1533 | HMDB0003072 | Organooxygen compounds | Alcohols and polyols |
| **103** | Sorbose | Sorbose 1 TMS | serum | 17.0 | 103 | 217 | 307 | 1545 | HMDB0001266 | Organooxygen compounds | Carbohydrates and carbohydrate conjugates |
|  |  | Sorbose 2 TMS | serum | 17.1 | 103 | 217 | 307 | 1550 |  |  |  |
| **104** | Glucuronic acid |  | GCF | 17.1 | 160 | 217 | 174 | 1575 | HMDB0000127 | Organooxygen compounds | Carbohydrates and carbohydrate conjugates |
| **105** | PYRANOSE 1 D-mannose 1/D-allose 1 |  | serum, saliva, GCF | 17.1 | 319 | 205 | 160 | 1580 | HMDB0000169 | Organooxygen compounds | Carbohydrates and carbohydrate conjugates |
| **106** | Tyrosine | Tyrosine 1 TMS | serum | 17.2 | 179 | 208 | 219 | 1562 | HMDB0000158 | Carboxylic acids and derivatives | Amino acids, peptides, and analogues |
|  |  | Tyrosine 2 TMS | serum | 17.7 | 218 | 280 | 354 | 1597 |  |  |  |
| **107** | PYRANOSE 2 (D-glucose/D (+) altrose 1/D (+) galactose 1/Talose 1) |  | serum, saliva, GCF | 17.3 | 319 | 205 | 160 | 1569 | HMDB0000169 | Organooxygen compounds | Carbohydrates and carbohydrate conjugates |
| **108** | 3-(4-hydroxyphenyl)lactic acid |  | saliva | 17.3 | 179 | 308 | 293 | 1570 | HMDB0303993 | Phenylpropanoic acids | nd |
| **110** | PYRANOSE 3 (D-glucose 2/D (+) altrose 2/D (+) galactose 2/Talose 2/Mannose 2/Allose 2) |  | serum, saliva | 17.5 | 205 | 319 | 160 | 1580 | HMDB0000122 | Organooxygen compounds | Carbohydrates and carbohydrate conjugates |
| **112** | Mannitol/Galactitol |  | serum, saliva | 17.6 | 319 | 205 | 217 | 1592 | HMDB0000765 | Organooxygen compounds | Carbohydrates and carbohydrate conjugates |
| **113** | Galacturonic acid | Galacturonic acid 1 TMS | serum | 17.7 | 333 | 160 | 189 | 1603 | HMDB0002545 | Organooxygen compounds | Carbohydrates and carbohydrate conjugates |
|  |  | Galacturonic acid 2 TMS | serum | 17.8 | 333 | 160 | 292 | 1625 |  |  |  |
| **114** | Sorbitol |  | saliva | 17.7 | 205 | 319 | 217 | 1602 | HMDB0000247 | Organooxygen compounds | Carbohydrates and carbohydrate conjugates |
| **115** | Tannic acid |  | saliva | 17.9 | 281 | 458 | 443 | 1616 | HMDB0258711 | Carboxylic acid esters | Polyphenols |
| **116** | 3-indoleacetic acid |  | serum, saliva | 18.0 | 202 | 319 | 203 | 1623 | HMDB0000197 | Indoles and derivatives | Indolyl carboxylic acids and derivatives |
| **117** | Glucopyranose |  | saliva | 18.1 | 204 | 191 | 217 | - | HMDB0000122 | Organooxygen compounds | Carbohydrates and carbohydrate conjugates |
| **118** | Glucosaminic acid |  | serum | 18.3 | 205 | 219 | 319 | 1657 | HMDB0341308 | Carboxylic acids and derivatives | Amino acids, peptides, and analogues |
| **119** | Galactonic acid |  | serum | 18.3 | 292 | 205 | 319 | 1670 | HMDB0000565 | Hydroxy acids and derivatives | Medium-chain hydroxy acids and derivatives |
| **120** | Scyllo-inositol |  | saliva | 18.6 | 139 | 129 | 157 | - | HMDB0006088 | Organooxygen compounds | Alcohols and polyols |
| **121** | Palmitoleic acid |  | serum | 18.6 | 311 | 117 | 129 | 1695 | HMDB0003229 | Fatty Acyls | Fatty acids and conjugates |
| **122** | Palmitic acid |  | serum, saliva | 18.8 | 313 | 117 | 129 | 1708 | HMDB0000220 | Fatty Acyls | Fatty acids and conjugates |
| **123** | Indole 3-propionic acid |  | serum | 19.2 | 202 | 333 | 203 | 1751 | HMDB0002302 | Indoles and derivatives | Indolyl carboxylic acids and derivatives |
| **124** | Myo-inositol |  | serum, saliva, GCF | 19.2 | 305 | 318 | 191 | 1768 | HMDB0000211 | Organooxygen compounds | Alcohols and polyols |
| **125** | Ribose-5-phosphate |  | saliva | 19.4 | 315 | 299 | 217 | 1784 | HMDB0001548 | Organooxygen compounds | Carbohydrates and carbohydrate conjugates |
| **126** | Tryptophan |  | serum | 20.3 | 202 | 73 | 291 | 1889 | HMDB0000929 | Indoles and derivatives | Indolyl carboxylic acids and derivatives |
| **127** | Linoleic acid |  | serum, GCF | 20.4 | 75 | 67 | 55 | 1885 | HMDB0000673 | Fatty Acyls | Lineolic acids and derivatives |
| **129** | Oleic acid |  | serum, saliva | 20.5 | 339 | 117 | 339 | 1892 | HMDB0000207 | Fatty Acyls | Fatty acids and conjugates |
| **130** | Trans-13-octadecenoic acid |  | serum | 20.5 | 129 | 117 | 145 | 1895 | HMDB0041480 | Fatty Acyls | Fatty acids and conjugates |
| **131** | Stearic acid |  | serum | 20.7 | 117 | 341 | 129 | 1907 | HMDB0000827 | Fatty Acyls | Fatty acids and conjugates |
| **132** | Arachidonic acid |  | serum | 21.8 | 79 | 91 | 67 | 2324 | HMDB0001043 | Fatty Acyls | Fatty acids and conjugates |
| **133** | 3,7-dihydroxyflavone |  | GCF | 23.4 | 383 | 184 | 311 | 2287 | HMDB0029522 | Flavonoids | O-methylated flavonoids |
| **134** | Lactulose | Lactulose 1 TMS | saliva | 23.7 | 204 | 217 | 361 | 2296 | HMDB0000740 | Organooxygen compounds | Carbohydrates and carbohydrate conjugates |
|  |  | Lactulose 2 TMS | saliva | 24.3 | 204 | 361 | 217 | 2376 |  |  |  |
| **135** | Eicosapentaenoic acid |  | serum | 24.0 | 361 | 217 | 437 | 2317 | HMDB0001999 | Fatty Acyls | Fatty acids and conjugates |
| **136** | Sucrose |  | saliva | 24.0 | 361 | 217 | 437 | 2313 | HMDB0000258 | Organooxygen compounds | Carbohydrates and carbohydrate conjugates |
| **137** | Lactose | Lactose 1 TMS | saliva | 24.4 | 204 | 217 | 361 | 2369 | HMDB0000186 | Organooxygen compounds | Carbohydrates and carbohydrate conjugates |
|  |  | Lactose 2 TMS | saliva | 24.5 | 204 | 217 | 361 | 2390 |  |  |  |
| **138** | Maltose | Maltose 1 TMS | serum, saliva, GCF | 24.8 | 361 | 204 | 217 | 2401 | HMDB0000163 | Organooxygen compounds | Carbohydrates and carbohydrate conjugates |
|  |  | Maltose 2 TMS | serum, saliva, GCF | 25.0 | 361 | 204 | 217 | 2433 |  |  |  |
| **139** | 2-Monostearin |  | GCF | 24.9 | 129 | 218 | 103 | - | HMDB0011131 | Glycerolipids | Monoradylglycerols |
| **140** | Maltitol |  | saliva | 25.5 | 361 | 204 | 217 | 2505 | HMDB0002928 | Fatty Acyls | Fatty acyl glycosides |
| **141** | Palatinitol (Synonym: Isomaltol ) | Palatinol 1 TMS | saliva | 26.1 | 204 | 361 | 319 | 2585 | HMDB0303336 | Organooxygen compounds | Carbonyl compounds |
|  |  | Palatinol 2 TMS | saliva | 26.2 | 204 | 361 | 217 | 2598 |  |  |  |
| **142** | α-Tocopherol, TMS derivative |  | serum | 28.0 | 502 | 237 | 236 | - | HMDB0001893 | Prenol lipids | Quinone and hydroquinone lipids |
| **142** | Cholesterol |  | serum, saliva, GCF | 28.2 | 329 | 73 | 368 | 2827 | HMDB0000067 | Steroids and steroid derivatives | Cholestane steroids |

**Table S1.** List of annotated metabolites (RT – retention time; HMDB – Human Metabolome Database; TI – target ion; QI 1 – first qualifier ion; QII 2 – second qualifier ion; RI – retention index). All compounds reported in the table are trimethylsilylated three methyl silylated (TMS), with the following number referring to the number of active hydrogens replaced by the TMS group, as reported in the Fiehn library.
