# Supplementary material for: Multi-matrix metabolomics in rare monogenic diabetes syndromes: Analysis of oral fluids and serum in carriers of pathogenic variants in the ALMS1/BBS genes
Source: Comput Struct Biotechnol J. 2025 Oct 22;27:4880–9. doi: 10.1016/j.csbj.2025.10.040 (PMC12648480; doi:10.1016/j.csbj.2025.10.040)
Supplement: Supplementary file 2 — Supplementary material [file mmc2.docx]

| **Type of sample** | **Number of statistically significant metabolites** | **Metabolites** |  |
| --- | --- | --- | --- |
| GCF Saliva Serum | 7 | Valine 3-Hydroxybutyric acid Alanine Threonine Urea Isoleucine Phenylalanine |  |
| GCF Saliva | 3 | Glutamic acid Serine Aspartic acid |  |
| GCF Serum | 3 | Glycine Pyruvic acid PYRANOSE D-mannose 1/D-allose 1 |  |
| Saliva Serum | 2 | Ornithine Quinic acid |  |
| GCF | 8 | 2-amino-2-methyl-1,3-propanediol Myo-inositol Ribose 2-Monostearin Malic acid 5-oxo-proline/pyroglutamic acid Lactic acid Maltose |  |
| Saliva | 21 | 3-aminoisobutyric acid Threitol Creatinine Scyllo-Inositol 1,3-diaminopropane Proline Beta-alanine Thymine Cadaverine Sorbitol Trans-4-hydroxy-L-proline Erythritol Gamma-aminobutyric acid (GABA) Putrescine Alpha-D-glucosamine phosphate Hypotaurine Pentanedioic acid Fucose Pyrophosphate Lysine 4-hydroxybutanoic acid |  |
| Serum | 30 | Tyrosine Trans-13-octadecenoic acid 2-hydroxybutyric acid Indole 3-propionic acid Alpha ketoglutaric acid Palmitoleic acid Fumaric acid PYRANOSE (D-glucose 2/D (+) altrose 2/D (+) galactose 2/Talose 2/Mannose 2/Allose 2) Cholesterol Palmitic acid Threonic acid Alpha-Tocopherol Citrulline Tryptophan Phosphoric acid Methionine N-methylalanine Linoleic acid PYRANOSE (D-glucose/D (+) altrose 1/D (+) galactose 1/Talose 1) Acetoacetate 3-methyl-2-oxobutanoic acid Sorbose Oleic acid Glycerol 1-phosphate Stearic acid Ethanolamine Mannitol Leucine Glycerol Galactitol |  |
|  |  |  |  |

**Table S2. Metabolites annotated in serum, saliva, and gingival crevicular fluid (GCF) by untargeted GC-MS analysis, as visualized using a Venn diagram.**
