# Supplementary material for: Multi-matrix metabolomics in rare monogenic diabetes syndromes: Analysis of oral fluids and serum in carriers of pathogenic variants in the ALMS1/BBS genes
Source: Comput Struct Biotechnol J. 2025 Oct 22;27:4880–9. doi: 10.1016/j.csbj.2025.10.040 (PMC12648480; doi:10.1016/j.csbj.2025.10.040)
Supplement: Supplementary file 3 — Supplementary material [file mmc3.docx]

| **Pathway name** | **Sample type** | **Match status** | **Expected** | **Hits** | **p-value** | **(- log (p))** | **Holm p** | **FDR** | **Impact** |
| --- | --- | --- | --- | --- | --- | --- | --- | --- | --- |
| Valine, leucine and isoleucine biosynthesis | GCF | 8 | 0.085 | 3 | 5.49E-05 | 4.26 | 4.39E-03 | 4.39E-03 | 0.54 |
| Alanine, aspartate and glutamate metabolism | GCF | 28 | 0.299 | 4 | 1.56E-04 | 3.81 | 1.23E-02 | 5.49E-03 | 0.42 |
| Glyoxylate and dicarboxylate metabolism | GCF | 32 | 0.342 | 4 | 2.67E-04 | 3.57 | 2.08E-02 | 5.49E-03 | 0.11 |
| Glycine, serine and threonine metabolism | GCF | 33 | 0.352 | 4 | 3.02E-04 | 3.52 | 2.32E-02 | 5.49E-03 | 0.47 |
| Arginine biosynthesis | GCF | 14 | 0.150 | 3 | 3.43E-04 | 3.46 | 2.61E-02 | 5.49E-03 | 0.12 |
| Glutathione metabolism | GCF | 28 | 0.299 | 3 | 2.81E-03 | 2.55 | 2.11E-01 | 3.75E-02 | 0.12 |
| Arginine biosynthesis | Saliva | 14 | 0.193 | 4 | 2.51E-05 | 4.60 | 2.01E-03 | 2.01E-03 | 0.19 |
| Arginine and proline metabolism | Saliva | 36 | 0.497 | 5 | 8.87E-05 | 4.05 | 7.01E-03 | 3.28E-03 | 0.39 |
| Valine, leucine and isoleucine biosynthesis | Saliva | 8 | 0.111 | 3 | 1.23E-04 | 3.91 | 9.58E-03 | 3.28E-03 | 0.75 |
| Glutathione metabolism | Saliva | 28 | 0.387 | 4 | 4.51E-04 | 3.35 | 3.47E-02 | 9.03E-03 | 0.03 |
| Glycine, serine and threonine metabolism | Saliva | 20 | 0.276 | 3 | 2.24E-03 | 2.65 | 1.71E-01 | 3.46E-02 | 0.05 |
| beta-Alanine biosynthesis | Saliva | 21 | 0.290 | 3 | 2.60E-03 | 2.59 | 1.95E-01 | 3.46E-02 | 0.40 |
| Alanine, aspartate and glutamate metabolism | Saliva | 28 | 0.387 | 3 | 6.00E-03 | 2.22 | 4.44E-01 | 6.86E-02 | 0.42 |
| Valine, leucine and isoleucine biosynthesis | serum | 8 | 0.121 | 4 | 2.68E-06 | 5.57 | 2.14E-04 | 2.14E-04 | 0.66 |
| Arginine biosynthesis | serum | 14 | 0.211 | 4 | 3.60E-05 | 4.44 | 2.85E-03 | 1.44E-03 | 0.06 |
| Phenylalanine, tyrosine and tryptophan biosynthesis | serum | 4 | 0.060 | 2 | 1.28E-03 | 2.89 | 1.00E-01 | 3.39E-02 | 1.00 |
| Biosynthesis of unsaturated fatty acids | serum | 36 | 0.543 | 4 | 1.70E-03 | 2.77 | 1.31E-01 | 3.39E-02 | 0.19 |
| Citrate cycle (TCA cycle) | serum | 20 | 0.302 | 3 | 2.90E-03 | 2.54 | 2.21E-01 | 4.64E-02 | 0.13 |
| Phenylalanine metabolism | serum | 8 | 0.121 | 2 | 5.77E-03 | 2.24 | 4.33E-01 | 6.85E-02 | 0.36 |

**Table S3.** Metabolic pathways are statistically significant based on pathway analysis in GCF, saliva, and serum.
