# Supplementary material for: Multi-matrix metabolomics in rare monogenic diabetes syndromes: Analysis of oral fluids and serum in carriers of pathogenic variants in the ALMS1/BBS genes
Source: Comput Struct Biotechnol J. 2025 Oct 22;27:4880–9. doi: 10.1016/j.csbj.2025.10.040 (PMC12648480; doi:10.1016/j.csbj.2025.10.040)
Supplement: Supplementary file 4 — Supplementary material [file mmc4.docx]

**Table S4**. Summary of statistical analysis (ANOVA/Kruskal–Wallis test) for GCF metabolite levels across study groups. Statistically significant p-values are in bold. Effect size metrics and 95% confidence intervals for group comparisons. ω² – global ANOVA ω²; ω²_ci_low / ω²_ci_high – 95% CI bounds for ω²; effsize – Cohen’s d for pairwise comparisons (IV vs III, IV vs ALMS+BBS, III vs ALMS+BBS); conf.low / conf.high – lower and upper 95% CI bounds for each Cohen’s d.

| **Subclass** | **Metabolites** | **HMDB** | **CV** | **ALMS+BBS vs. III vs. IV** | **Omega2** | **Omega2_ci_low** | **Omega2_ci_high** |  | **post-hoc** | | | | | | | | | |  |  |  |  |
| --- | --- | --- | --- | --- | --- | --- | --- | --- | --- | --- | --- | --- | --- | --- | --- | --- | --- | --- | --- | --- | --- | --- |
|  |  |  |  |  |  |  |  | **IV vs. III** | **% of changes** | effsize_IV vs. III | conf.low_IV vs. III | **conf.high_IV vs. III** | **IV vs. ALMS+BBS** | **% of changes** | **effsize_IV vs. ALMS+BBS** | **conf.low_IV vs. ALMS+BBS** | conf.high_IV vs. ALMS+BBS | **III vs. ALMS+BBS** | **% of changes** | effsize_III vs. ALMS+BBS | conf.low_III vs. ALMS+BBS | conf.high_III vs. ALMS+BBS |
| Alcohols and polyols | Myo-inositol | HMDB0000211 | 9.7 | 1.41E-01 | 3.98E-02 | 0.00E+00 | 2.13E-01 | 1.29E-01 | 11.8 | -0.31 | -0.88 | 0.25 | 1.58E-01 | -11.3 | 0.36 | -0.10 | 0.87 | **3.51E-02** | **-20.7** | **0.67** | **0.12** | **1.21** |
| Alpha hydroxy acids and derivatives | Lactic acid | HMDB0000190 | 7.1 | 1.77E-01 | 8.45E-02 | 0.00E+00 | 2.73E-01 | 1.16E-01 | -13.7 | 0.45 | -0.09 | 1.04 | 5.36E-02 | -19.6 | 0.59 | 0.09 | 1.10 | 3.38E-01 | -6.7 | 0.19 | -0.38 | 0.73 |
| Alpha-keto acids and derivatives | Pyruvic acid | HMDB0000243 | 26.9 | **8.99E-05** | **1.86E-01** | **8.01E-02** | **3.52E-01** | 2.49E-01 | -18.5 | 0.22 | -0.32 | 0.80 | **1.55E-04** | **113.0** | **-0.92** | **-1.46** | **-0.46** | **9.77E-05** | **161.3** | **-1.10** | **-1.75** | **-0.67** |
| Amines | 2-amino-2-methyl-1.3-propanediol | HMDB0244975 | 19.9 | 1.41E-01 | 4.45E-02 | 0.00E+00 | 2.15E-01 | **4.70E-02** | **-17.5** | **0.53** | **-0.07** | **1.18** | 5.91E-02 | -16.1 | 0.59 | 0.09 | 1.13 | 4.75E-01 | 1.6 | -0.05 | -0.71 | 0.56 |
| Amino acids, peptides and analogues | Valine | HMDB0000883 | 7.2 | **4.02E-07** | **4.65E-01** | **3.44E-01** | **6.19E-01** | 4.76E-01 | 0.1 | 0.00 | -0.60 | 0.57 | **2.42E-07** | **85.4** | **-1.64** | **-2.28** | **-1.20** | **3.12E-06** | **85.2** | **-1.68** | **-2.35** | **-1.22** |
|  | Alanine | HMDB0000161 | 14.9 | **7.30E-06** | **3.55E-01** | **2.04E-01** | **5.60E-01** | 2.04E-01 | -10.8 | 0.28 | -0.30 | 0.88 | **2.27E-05** | **75.7** | **-1.22** | **-1.82** | **-0.70** | **7.84E-06** | **97.0** | **-1.41** | **-2.01** | **-0.93** |
|  | Isoleucine | HMDB00172 | 12.1 | **2.25E-07** | **4.49E-01** | **3.41E-01** | **6.06E-01** | 2.02E-01 | 15.1 | -0.31 | -0.94 | 0.34 | **1.04E-08** | **130.8** | **-1.80** | **-2.39** | **-1.39** | **1.24E-05** | **100.5** | **-1.43** | **-2.09** | **-0.98** |
|  | Serine | HMDB00187 | 12.2 | **8.99E-05** | **2.62E-01** | **1.43E-01** | **4.34E-01** | 2.84E-01 | -7.7 | 0.21 | -0.38 | 0.83 | **1.28E-04** | **52.4** | **-1.15** | **-1.66** | **-0.73** | **1.29E-04** | **65.1** | **-1.28** | **-1.82** | **-0.84** |
|  | Threonine | HMDB0000167 | 17.8 | **5.37E-05** | **2.46E-01** | **1.17E-01** | **4.27E-01** | 4.92E-01 | -4.3 | 0.10 | -0.52 | 0.64 | **4.43E-05** | **54.8** | **-1.12** | **-1.80** | **-0.57** | **1.39E-04** | **61.7** | **-1.26** | **-1.94** | **-0.77** |
|  | Glycine | HMDB00123 | 5.3 | **5.62E-06** | **3.12E-01** | **1.91E-01** | **4.73E-01** | 3.56E-01 | -7.3 | 0.17 | -0.41 | 0.80 | **8.84E-06** | **55.1** | **-1.33** | **-1.95** | **-0.83** | **7.81E-06** | **67.4** | **-1.53** | **-2.26** | **-1.00** |
|  | Glutamic acid | HMDB0000148 | 18.1 | **5.67E-06** | **2.88E-01** | **1.61E-01** | **5.07E-01** | 3.11E-01 | 18.5 | -0.19 | -0.70 | 0.46 | **1.56E-06** | **205.1** | **-1.26** | **-1.74** | **-0.94** | **9.06E-05** | **157.5** | **-1.08** | **-1.67** | **-0.57** |
|  | Phenylalanine | HMDB0000159 | 25.6 | **2.72E-07** | **4.25E-01** | **3.02E-01** | **6.11E-01** | 2.40E-01 | 32.5 | -0.33 | -1.00 | 0.23 | **2.90E-08** | **259.9** | **-1.74** | **-2.35** | **-1.28** | **1.39E-05** | **171.5** | **-1.43** | **-2.03** | **-0.93** |
|  | 5-oxo-proline/pyroglutamic acid | HMDB0000267 | 11.1 | **2.57E-04** | **2.16E-01** | **9.70E-02** | **3.85E-01** | 4.30E-01 | 1.3 | -0.03 | -0.68 | 0.51 | **1.42E-04** | **49.9** | **-1.15** | **-1.77** | **-0.66** | **7.41E-04** | **48.0** | **-1.15** | **-1.84** | **-0.66** |
|  | Aspartic acid | HMDB0000191 | 13.1 | **5.22E-05** | **1.68E-01** | **7.07E-02** | **3.44E-01** | 4.03E-01 | 10.4 | -0.12 | -0.56 | 0.64 | **2.30E-05** | **149.7** | **-0.99** | **-1.40** | **-0.66** | **2.41E-04** | **126.1** | **-0.84** | **-1.40** | **-0.34** |
| Beta hydroxy acids and derivatives | 3-Hydroxybutyric acid | HMDB0000011 | 23.5 | **5.22E-05** | **1.55E-01** | **8.30E-02** | **3.45E-01** | **1.23E-02** | **-55.0** | **0.73** | **0.34** | **1.16** | **5.58E-03** | **58.0** | **-0.55** | **-1.10** | **-0.05** | **4.43E-06** | **251.4** | **-1.36** | **-1.92** | **-1.04** |
|  | Malic acid | HMDB0031518 | 14.8 | 8.58E-01 | 0.00E+00 | 0.00E+00 | 8.57E-02 | 4.37E-01 | 2.8 | -0.07 | -0.62 | 0.57 | 5.17E-01 | -3.5 | 0.08 | -0.39 | 0.59 | 9.08E-01 | -6.2 | 0.14 | -0.44 | 0.71 |
| Carbohydrates and carbohydrate conjugates | PYRANOSE D-mannose 1/D-allose 1 | HMDB00169 | 39.4 | 4.15E-01 | 4.03E-02 | 0.00E+00 | 1.58E-01 | 2.49E-01 | 40.5 | -0.47 | -1.04 | 0.15 | 4.82E-01 | 7.8 | -0.11 | -0.60 | 0.41 | 1.28E-01 | -23.3 | 0.36 | -0.19 | 0.95 |
|  | Maltose | HMDB0000163 | 30.0 | **1.61E-02** | **2.40E-03** | **0.00E+00** | **1.83E-01** | 3.30E-01 | -37.5 | 0.23 | -0.60 | 0.52 | **7.11E-03** | **-63.3** | **0.39** | **0.23** | **0.86** | **8.71E-03** | **-41.3** | **0.79** | **0.24** | **1.34** |
|  | Ribose | HMDB0000283 | 29.1 | 5.32E-02 | 4.38E-02 | 0.00E+00 | 2.31E-01 | 4.99E-01 | 26.2 | -0.21 | -0.65 | 0.55 | **2.30E-02** | **97.4** | **-0.67** | **-1.05** | **-0.32** | **2.33E-02** | **56.5** | **-0.40** | **-1.05** | **0.15** |
| Monoradylglycerols | 2-Monostearin | HMDB11131 | 26.8 | **4.58E-02** | **1.24E-02** | **0.00E+00** | **1.80E-01** | 1.45E-01 | 10.9 | -0.14 | -0.76 | 0.43 | 5.12E-02 | -30.0 | 0.35 | -0.16 | 0.89 | **1.03E-02** | **-36.9** | **0.51** | **-0.02** | **1.14** |
| Ureas | Urea | HMDB00294 | 20.1 | **2.33E-06** | **1.70E-01** | **5.87E-02** | **3.60E-01** | 3.55E-01 | 15.4 | -0.17 | -0.77 | 0.46 | **3.70E-06** | **-74.3** | **1.00** | **0.37** | **1.77** | **3.62E-06** | **-77.7** | **1.06** | **0.46** | **1.84** |
