# Supplementary material for: Multi-matrix metabolomics in rare monogenic diabetes syndromes: Analysis of oral fluids and serum in carriers of pathogenic variants in the ALMS1/BBS genes
Source: Comput Struct Biotechnol J. 2025 Oct 22;27:4880–9. doi: 10.1016/j.csbj.2025.10.040 (PMC12648480; doi:10.1016/j.csbj.2025.10.040)
Supplement: Supplementary file 5 — Supplementary material [file mmc5.docx]

**Table S5**. Summary of statistical analysis (ANOVA/Kruskal–Wallis test) for serum metabolite levels across study groups. Statistically significant p-values are in bold. Effect size metrics and 95% confidence intervals for group comparisons. ω² – global ANOVA ω²; ω²_ci_low / ω²_ci_high – 95% CI bounds for ω²; effsize – Cohen’s d for pairwise comparisons (IV vs III, IV vs ALMS+BBS, III vs ALMS+BBS); conf.low / conf.high – lower and upper 95% CI bounds for each Cohen’s d.

| **Subclass** | **Metabolites** | **HMDB** | **CV** | **ALMS+BBS vs. III vs. IV** | **Omega2** | **Omega2_ci_low** | **Omega2_ci_high** |  | **post-hoc** | | | | | | | | | | | | | |
| --- | --- | --- | --- | --- | --- | --- | --- | --- | --- | --- | --- | --- | --- | --- | --- | --- | --- | --- | --- | --- | --- | --- |
|  |  |  |  |  |  |  |  | **IV vs. III** | **% of changes** | **conf.low_IV vs. III** | **conf.high_IV vs. III** | **effsize_IV vs. ALMS+BBS** | **IV vs. ALMS+BBS** | **% of changes** | **effsize_IV vs. ALMS+BBS** | **conf.low_IV vs. ALMS+BBS** | **conf.high_IV vs. ALMS+BBS** | **III vs. ALMS+BBS** | **% of changes** | **effsize_III vs. ALMS+BBS** | **conf.low_III vs. ALMS+BBS** | **conf.high_III vs. ALMS+BBS** |
| Alcohols and polyols | Quinic acid | HMDB0003072 | 6.2 | **4.54E-02** | **6.72E-02** | **2.94E-02** | **2.05E-01** | **4.38E-02** | 31.8 | -0.9 | 0.3 | 0.5 | 1.03E-01 | -46.4 | 0.5 | 0.0 | 0.9 | **2.87E-03** | -59.4 | 0.819691496 | 0.39 | 1.3 |
| Alpha-keto acids and derivatives | Pyruvic acid | HMDB0000243 | 6.7 | 1.46E-01 | 7.25E-02 | 5.85E-03 | 2.65E-01 | 9.39E-02 | 19.2 | -1.1 | 0.1 | -0.7 | **2.27E-02** | 29.0 | -0.7 | -1.2 | -0.2 | 2.65E-01 | 8.2 | -0.204952014 | -0.76 | 0.39 |
| Amines | Ethanolamine | HMDB0000149 | 8.3 | 1.14E-01 | 6.55E-02 | 2.30E-03 | 2.17E-01 | 5.57E-02 | 11.8 | -1.1 | 0.1 | 0.3 | 1.85E-01 | -5.9 | 0.3 | -0.2 | 0.8 | **1.17E-02** | -15.8 | 0.853213006 | 0.38 | 1.33 |
| Amino acids, peptides and analogues | Valine | HMDB0000883 | 15.1 | 1.53E-01 | 7.10E-02 | 0.00E+00 | 2.46E-01 | **4.23E-02** | 16.6 | -1.1 | 0.0 | 0.1 | 3.48E-01 | -4.2 | 0.1 | -0.4 | 0.6 | **3.01E-02** | -17.8 | 0.690915328 | 0.19 | 1.21 |
| Amino acids, peptides and analogues | Isoleucine | HMDB00172 | 13.6 | 1.37E-01 | 5.94E-02 | 0.00E+00 | 2.20E-01 | 2.86E-02 | 18.5 | -1.0 | 0.0 | 0.1 | 3.78E-01 | -4.9 | 0.1 | -0.4 | 0.6 | **2.31E-02** | -19.8 | 0.680760311 | 0.19 | 1.15 |
| Amino acids, peptides and analogues | Threonine | HMDB0000167 | 19.4 | 1.04E-01 | 4.35E-02 | 0.00E+00 | 2.10E-01 | 1.95E-01 | 6.7 | -0.9 | 0.4 | 0.5 | **4.24E-02** | -15.6 | 0.5 | -0.1 | 1.0 | **1.31E-02** | -21.0 | 0.737459584 | 0.2 | 1.41 |
| Amino acids, peptides and analogues | Glycine | HMDB00123 | 12.6 | 1.04E-01 | 6.24E-02 | 0.00E+00 | 2.18E-01 | 2.58E-01 | -3.8 | -0.4 | 0.8 | 0.6 | **1.07E-02** | -16.8 | 0.6 | 0.1 | 1.2 | 5.64E-02 | -13.5 | 0.481650676 | -0.06 | 1.01 |
| Amino acids, peptides and analogues | Methionine | HMDB0000696 | 22.0 | 5.27E-02 | 6.92E-02 | 3.90E-03 | 2.31E-01 | 1.10E-01 | 7.6 | -0.8 | 0.3 | 0.5 | **4.71E-02** | -17.8 | 0.5 | 0.0 | 1.0 | **4.61E-03** | -23.6 | 0.906080313 | 0.44 | 1.46 |
| Amino acids, peptides and analogues | Phenylalanine | HMDB0000159 | 16.5 | 2.36E-01 | 6.80E-02 | 0.00E+00 | 2.27E-01 | 1.01E-01 | 18.3 | -1.1 | 0.0 | 0.2 | 2.52E-01 | -2.0 | 0.2 | -0.3 | 0.7 | **4.73E-02** | -4.0 | 0.738162666 | 0.25 | 1.25 |
| Amino acids, peptides and analogues | Tyrosine | HMDB00158 | 12.1 | **8.31E-03** | **1.73E-01** | **8.60E-02** | **3.45E-01** | **1.20E-02** | 22.5 | -1.3 | -0.2 | 0.5 | 6.00E-02 | -14.4 | 0.5 | 0.0 | 1.0 | **1.44E-04** | -30.1 | 1.244093844 | 0.73 | 1.88 |
| Amino acids, peptides and analogues | Leucine | HMDB0000687 | 14.9 | 1.14E-01 | 5.89E-02 | 0.00E+00 | 2.18E-01 | **1.71E-02** | 21.0 | -1.1 | 0.0 | 0.1 | 4.40E-01 | -3.4 | 0.1 | -0.4 | 0.6 | **1.93E-02** | -20.2 | 0.731625811 | 0.29 | 1.22 |
| Amino acids, peptides and analogues | Ornithine | HMDB0000214 | 15.4 | 1.51E-01 | 5.94E-02 | 0.00E+00 | 2.20E-01 | 1.44E-01 | 17.0 | -1.1 | 0.1 | 0.3 | 1.19E-01 | -11.4 | 0.3 | -0.1 | 0.9 | **2.31E-02** | -24.2 | 0.820444118 | 0.3 | 1.49 |
| Beta hydroxy acids and derivatives | 2-hydroxybutyric acid | HMDB00011 | 5.7 | **1.50E-02** | **1.47E-01** | **3.46E-02** | **3.76E-01** | 1.66E-01 | 20.5 | -0.8 | 0.2 | -0.9 | **6.99E-04** | 51.0 | -0.9 | -1.4 | -0.5 | **2.17E-02** | 25.3 | -0.427885103 | -1.13 | 0.16 |
| Beta hydroxy acids and derivatives | 3-Hydroxybutyric acid | HMDB0000011 | 5.7 | **4.19E-05** | **2.07E-01** | **7.28E-02** | **4.08E-01** | 9.13E-02 | -6.1 | -0.5 | 0.9 | -1.0 | **8.19E-05** | 173.4 | -1.0 | -1.5 | -0.6 | **1.44E-06** | 191.3 | -0.943637035 | -1.67 | -0.5 |
| Carbohydrates and carbohydrate conjugates | PYRANOSE D-mannose 1/D-allose 1 | HMDB00169 | 4.8 | 1.99E-01 | 3.55E-02 | 0.00E+00 | 1.73E-01 | **4.16E-02** | 20.0 | -1.3 | -0.2 | -0.5 | 8.89E-02 | 14.7 | -0.5 | -0.9 | 0.0 | 1.97E-01 | -4.4 | 0.164939757 | -0.35 | 0.75 |
| Carbohydrates and carbohydrate conjugates | Threonic acid | HMDB0000943 | 4.1 | 2.68E-01 | 0.00E+00 | 0.00E+00 | 1.06E-01 | 7.69E-02 | 5.6 | -0.2 | 0.8 | 0.0 | 8.02E-02 | -0.9 | 0.0 | -0.5 | 0.5 | 4.71E-01 | 20.7 | -0.374624763 | -0.88 | 0.14 |
| Carbohydrates and carbohydrate conjugates | Sorbose | HMDB0001266 | 18.6 | 1.51E-01 | 4.55E-02 | 7.77E-03 | 1.76E-01 | 1.87E-01 | -15.0 | -0.2 | 0.9 | 0.5 | **2.25E-02** | -21.3 | 0.5 | 0.1 | 1.0 | 1.53E-01 | -7.5 | 0.210083997 | -0.33 | 0.75 |
| Carbohydrates and carbohydrate conjugates | Mannitol | HMDB0000765 | 8.1 | **1.53E-02** | **9.55E-04** | **0.00E+00** | **1.55E-01** | **4.89E-02** | 22.9 | -0.8 | 0.4 | 0.3 | **4.19E-02** | -60.4 | 0.3 | -0.1 | 0.7 | **8.37E-04** | -67.7 | 0.54059784 | 0.16 | 0.97 |
| Carbohydrates and carbohydrate conjugates | Glycerol | HMDB0000131 | 5.1 | 7.76E-01 | 4.06E-02 | 0.00E+00 | 2.37E-01 | 5.17E-01 | 22.3 | -0.9 | 0.3 | -0.3 | 2.73E-01 | 21.6 | -0.3 | -0.8 | 0.2 | 4.40E-01 | -0.5 | 0.008315241 | -0.56 | 0.55 |
| Carbohydrates and carbohydrate conjugates | Galactitol | HMDB0000107 | 9.9 | **4.22E-02** | **0.00E+00** | **0.00E+00** | **1.51E-01** | **4.07E-02** | 24.3 | -0.7 | 0.4 | 0.3 | 1.02E-01 | -60.6 | 0.3 | -0.2 | 0.6 | **2.49E-03** | -68.3 | 0.514051774 | 0.1 | 0.92 |
| Dicarboxylic acids and derivatives | Fumaric acid | HMDB00134 | 18.1 | **1.53E-02** | **1.21E-01** | **2.60E-02** | **2.95E-01** | 1.28E-01 | -8.5 | -0.2 | 1.0 | 0.9 | **8.70E-04** | -20.6 | 0.9 | 0.5 | 1.6 | **3.72E-02** | -13.1 | 0.58539818 | 0.05 | 1.23 |
| Fatty acid esters | 3-methyl-2-oxobutanoic acid | HMDB0030027 | 5.9 | 3.01E-01 | 3.16E-02 | 0.00E+00 | 1.90E-01 | 2.40E-01 | 5.6 | -0.9 | 0.3 | -0.3 | 6.75E-02 | 8.6 | -0.3 | -1.0 | 0.2 | 2.12E-01 | 2.9 | -0.132056673 | -0.78 | 0.4 |
| Fatty acids and conjugates | Trans-13-octadecenoic acid | HMDB0041480 | 7.7 | **4.78E-03** | **2.26E-01** | **8.65E-02** | **4.27E-01** | 3.66E-01 | -10.3 | -0.4 | 0.7 | -0.9 | **4.18E-04** | 67.2 | -0.9 | -1.4 | -0.4 | **7.08E-04** | 86.4 | -1.067714489 | -1.68 | -0.59 |
| Fatty acids and conjugates | Stearic acid | HMDB00827 | 7.0 | 1.14E-01 | 7.39E-02 | 0.00E+00 | 2.51E-01 | 3.45E-01 | -3.4 | -0.5 | 0.7 | -0.6 | **2.36E-02** | 22.3 | -0.6 | -1.2 | -0.1 | **2.67E-02** | 26.7 | -0.683770417 | -1.36 | -0.13 |
| Fatty acids and conjugates | Palmitoleic acid | HMDB0003229 | 6.2 | **1.50E-02** | **1.16E-01** | **3.38E-02** | **3.32E-01** | 3.81E-01 | -12.3 | -0.5 | 0.7 | -0.7 | **2.15E-03** | 103.3 | -0.7 | -1.1 | -0.3 | **3.70E-03** | 132.0 | -0.790148914 | -1.24 | -0.44 |
| Fatty acids and conjugates | Palmitic acid | HMDB0000220 | 3.9 | **1.50E-02** | **1.14E-01** | **1.32E-02** | **2.87E-01** | 4.15E-01 | -3.7 | -0.5 | 0.7 | -0.7 | **3.65E-03** | 37.4 | -0.7 | -1.4 | -0.2 | **2.15E-03** | 42.8 | -0.824768466 | -1.61 | -0.24 |
| Fatty acids and conjugates | Oleic acid | HMDB0000207 | 3.4 | **4.78E-03** | **1.90E-01** | **7.45E-02** | **3.67E-01** | 3.35E-01 | -7.8 | -0.5 | 0.7 | -0.9 | **5.60E-04** | 62.4 | -0.9 | -1.6 | -0.4 | **6.73E-04** | 76.2 | -1.07209846 | -1.82 | -0.53 |
| Gamma-keto acids and derivatives | Alpha ketoglutaric acid | HMDB0000208 | 7.8 | **1.16E-02** | **1.73E-01** | **7.64E-02** | **3.87E-01** | 5.23E-02 | 18.1 | -1.0 | 0.0 | -1.0 | **2.53E-04** | 26.1 | -1.0 | -1.6 | -0.6 | 6.00E-02 | 6.7 | -0.228018777 | -0.98 | 0.29 |
| Glycerophosphates | Glycerol 1-phosphate | HMDB0000126 | 7.4 | **4.22E-02** | **9.50E-02** | **1.87E-02** | **2.41E-01** | 3.80E-01 | -2.8 | -0.5 | 0.6 | 0.7 | **6.77E-03** | -16.9 | 0.7 | 0.3 | 1.2 | **1.06E-02** | -14.5 | 0.812307818 | 0.25 | 1.47 |
| Indolyl carboxylic acids and derivatives | Tryptophan | HMDB0000929 | 10.9 | 1.10E-01 | 6.48E-02 | 0.00E+00 | 2.30E-01 | **3.08E-02** | 15.8 | -1.2 | 0.1 | 0.2 | 2.67E-01 | -6.4 | 0.2 | -0.3 | 0.7 | **1.12E-02** | -19.2 | 0.902141281 | 0.38 | 1.51 |
| Indolyl carboxylic acids and derivatives | Indole 3-propionic acid | HMDB0002302 | 4.5 | 1.46E-01 | 4.40E-02 | 9.88E-03 | 1.63E-01 | **2.03E-02** | -54.4 | 0.3 | 1.1 | 0.0 | 4.70E-01 | 0.9 | 0.0 | -0.5 | 0.5 | **4.02E-02** | 121.3 | -0.668462607 | -1.13 | -0.32 |
| Lineolic acids and derivatives | Linoleic acid | HMDB0000673 | 6.6 | **1.53E-02** | **1.06E-01** | **2.19E-02** | **2.87E-01** | 4.50E-01 | -4.8 | -0.5 | 0.6 | -0.7 | **3.73E-03** | 39.0 | -0.7 | -1.3 | -0.2 | **2.93E-03** | 46.1 | -0.795909667 | -1.55 | -0.21 |
| Short-chain keto acids and derivatives | Acetoacetate | HMDB0304256 | 12.0 | **4.19E-05** | **2.38E-01** | **9.43E-02** | **4.60E-01** | 2.74E-01 | -3.0 | -0.5 | 0.7 | -0.9 | **1.08E-05** | 122.1 | -0.9 | -1.5 | -0.6 | **7.28E-06** | 129.0 | -0.871319413 | -1.47 | -0.52 |
| Ureas | Urea | HMDB00294 | 4.3 | 1.46E-01 | 0.00E+00 | 0.00E+00 | 1.64E-01 | 1.26E-01 | 10.0 | -1.0 | 0.2 | 0.1 | 1.26E-01 | -4.3 | 0.1 | -0.3 | 0.7 | **2.21E-02** | -12.9 | 0.413814508 | -0.1 | 1.17 |
