# Supplementary figures and images for: Multi-matrix metabolomics in rare monogenic diabetes syndromes: Analysis of oral fluids and serum in carriers of pathogenic variants in the ALMS1/BBS genes
Source: Comput Struct Biotechnol J. 2025 Oct 22;27:4880–9. doi: 10.1016/j.csbj.2025.10.040 (PMC12648480; doi:10.1016/j.csbj.2025.10.040)

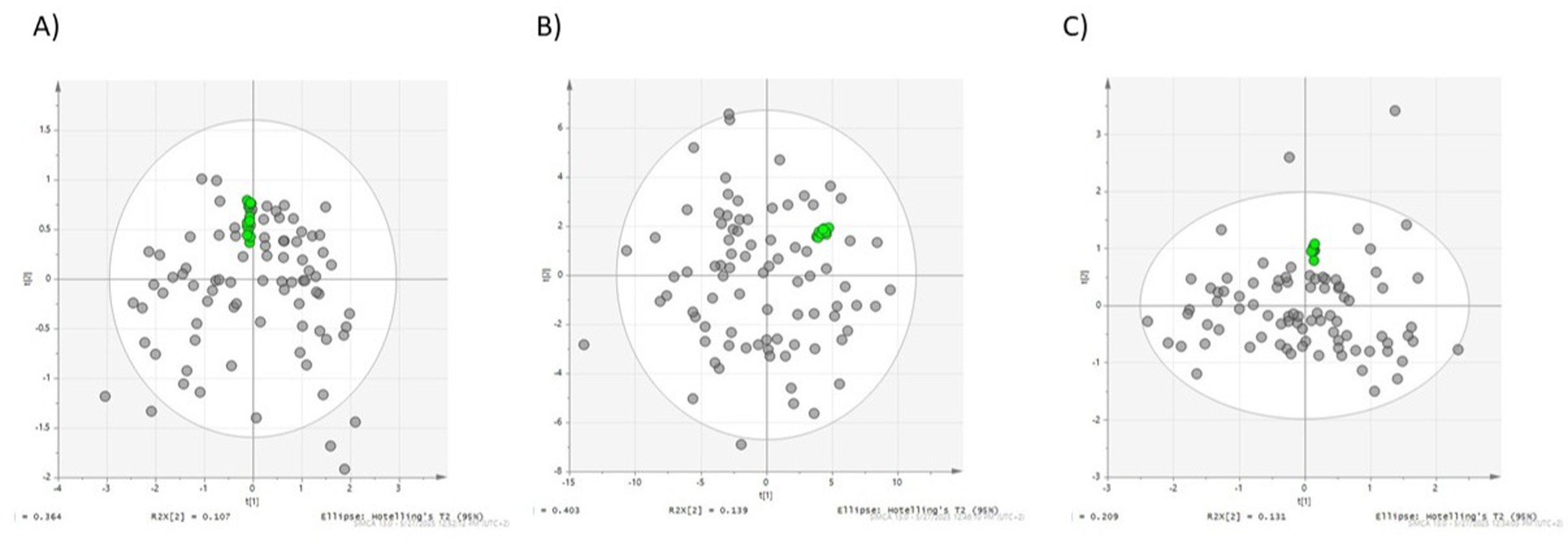

Supplement: Supplementary file 6 — Supplementary material Figure S1. PCA analysis showing QC sample distribution (green dots) in A) GCF (data were logarithmized, univariate scaling was applied, R2 = 0.472), B) saliva (data were logarithmized, Pareto scaling was applied, R2 = 0.784), and C) serum GCF (data were logarithmized, univariate scaling was applied, R2 = 0.541) [file mmc6.jpg]

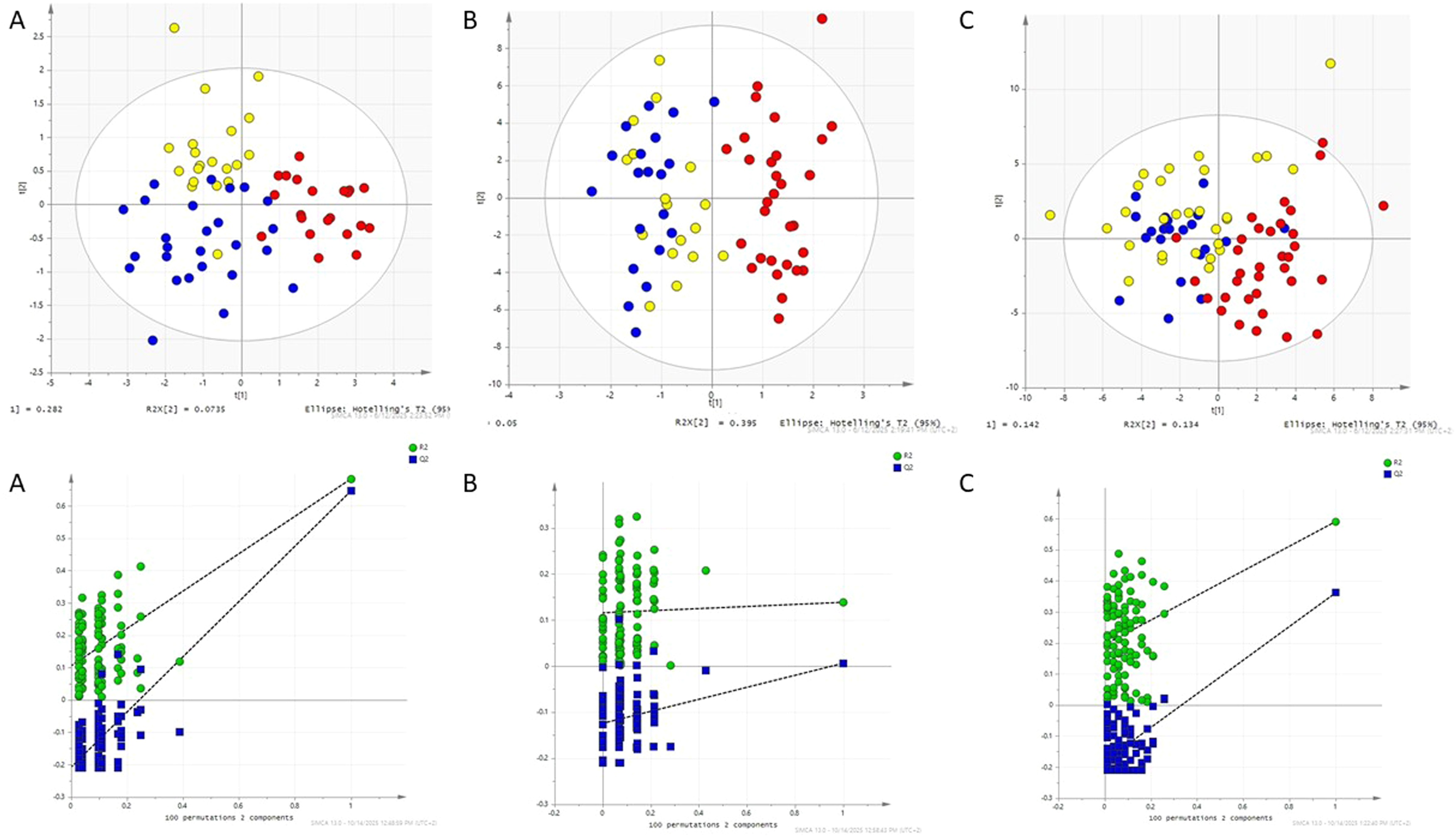

Supplement: Supplementary file 7 — Supplementary material Figure S2. Partial least squares discriminant analysis (PLS-DA) score plots of the serum fingerprints of ALMS/BBS patients (red dots), participants with obesity (blue dots) and the control group (yellow dots). A) GCF: R2 = 0.608, Q2 = 0.464 (p = 1.31 ×10 −3), B) saliva, R2 = 0.473, Q2 = 0.247 (p = 4.1 ×10 −2), C) serum R2 = 0.453, Q2 = 0.343 (p = 3.74 ×10 −5) [file mmc7.jpg]

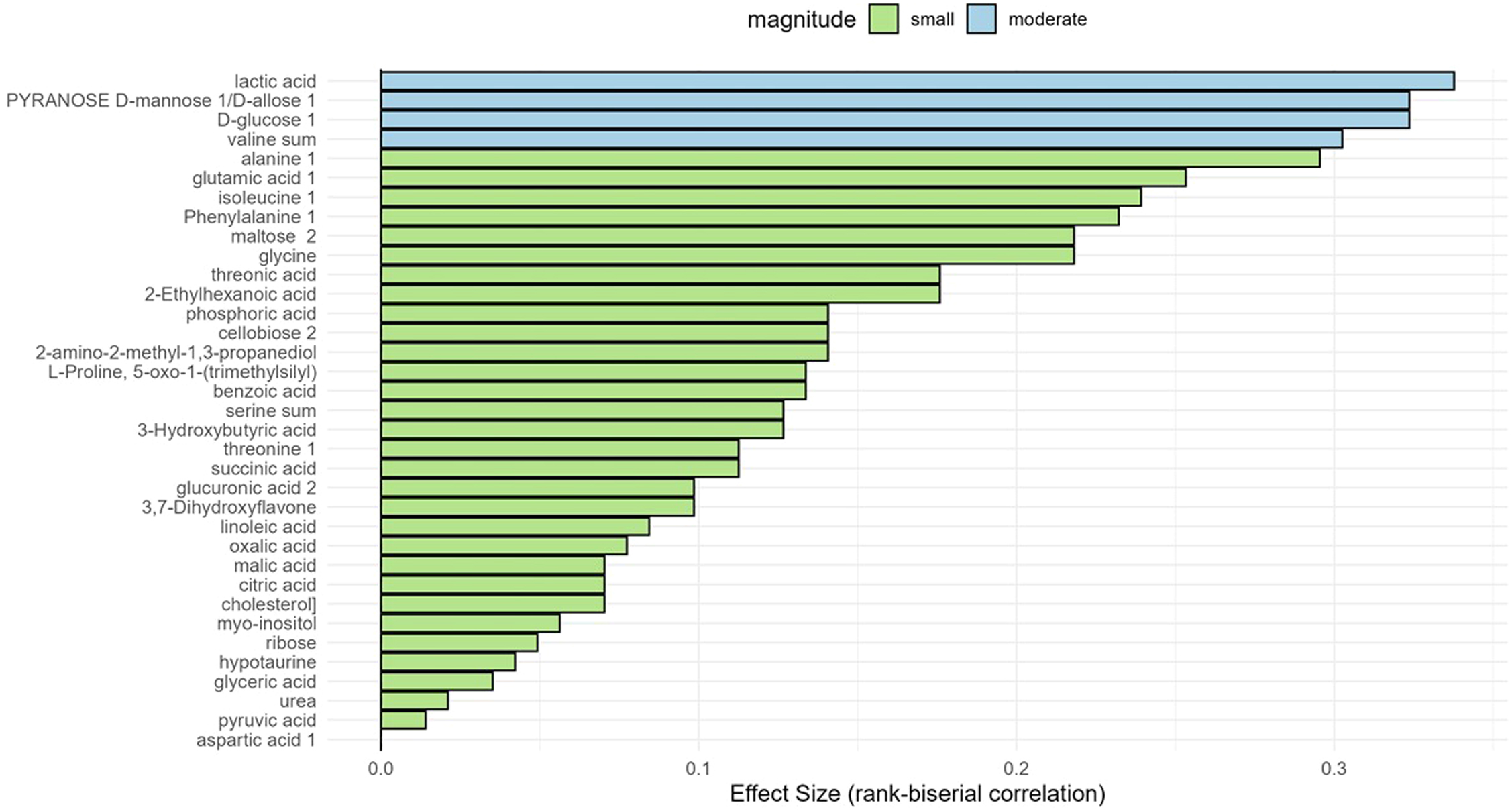

Supplement: Supplementary file 8 — Supplementary material Figure S3. Effect sizes (rank-biserial correlations) for metabolites differentiating ALMS+BBS homozygous and heterozygous groups in GCF [file mmc8.jpg]

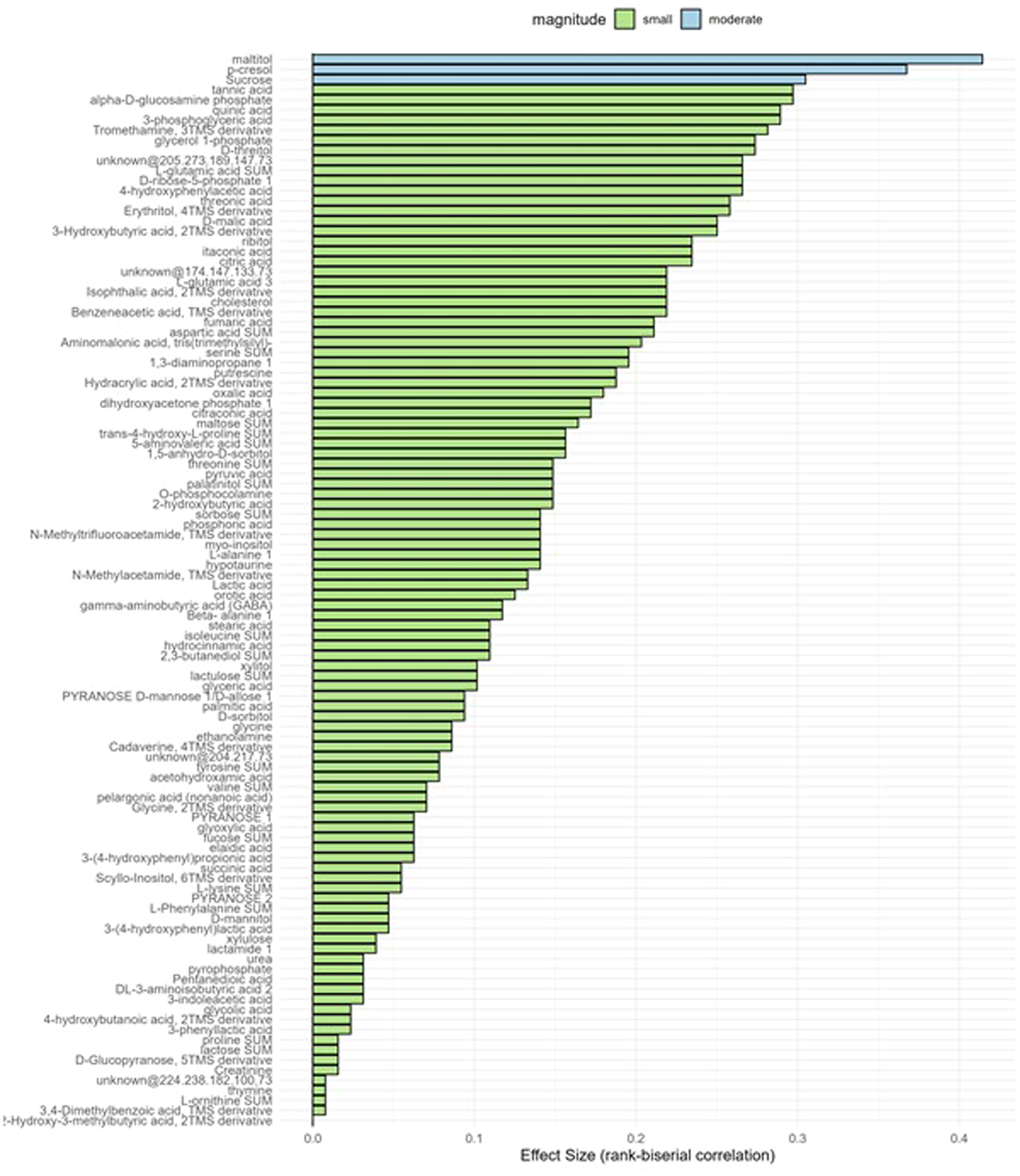

Supplement: Supplementary file 9 — Supplementary material Figure S4. Effect sizes (rank-biserial correlations) for metabolites differentiating ALMS+BBS homozygous and heterozygous groups in saliva [file mmc9.jpg]

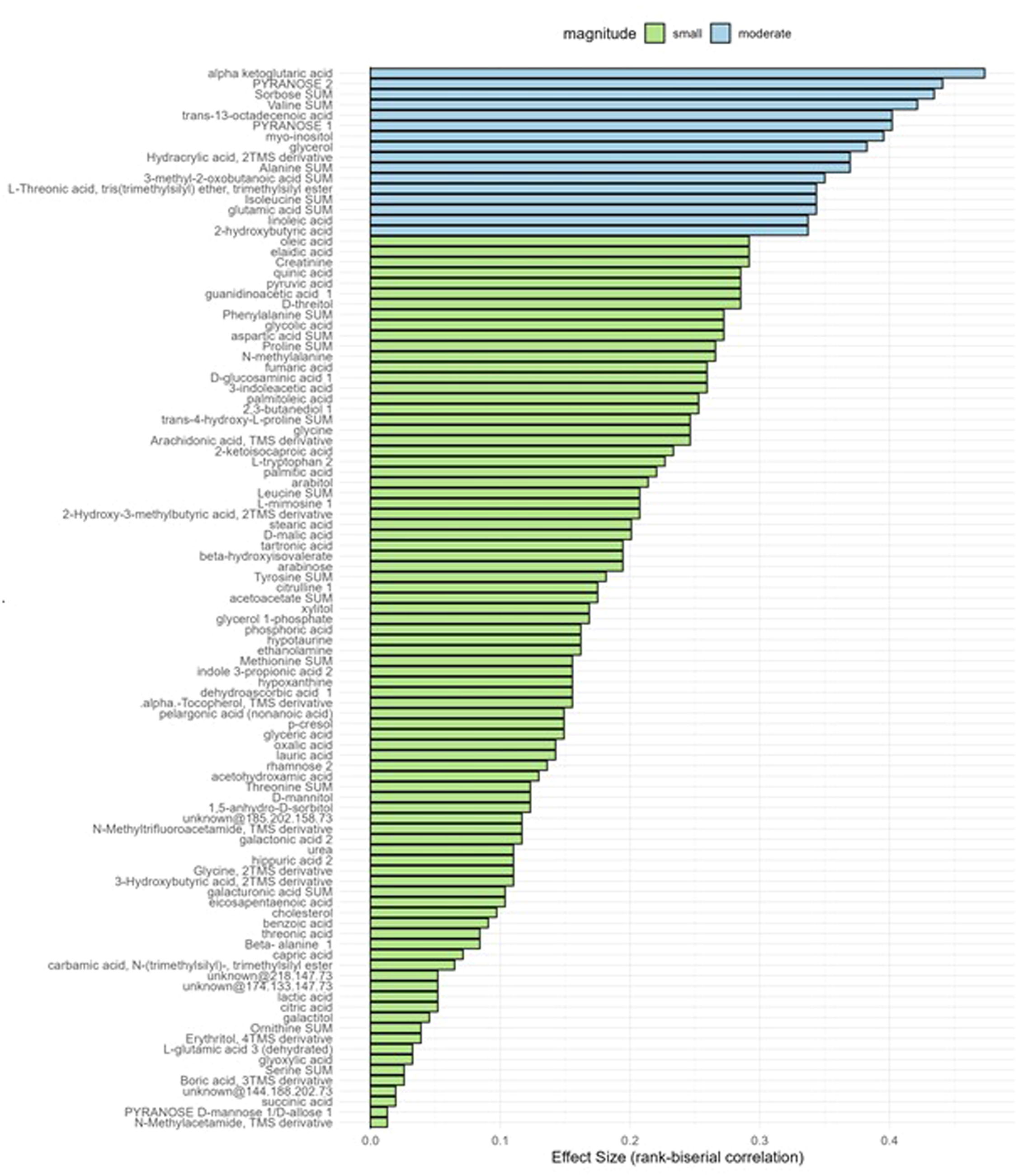

Supplement: Supplementary file 10 — Supplementary material Figure S5. Effect sizes (rank-biserial correlations) for metabolites differentiating ALMS+BBS homozygous and heterozygous groups in serum [file mmc10.jpg]
